# The evolutionary history of *mariner* elements in stalk-eyed flies reveals the horizontal transfer of transposons from insects into the genome of the cnidarian *Hydra vulgaris*

**DOI:** 10.1371/journal.pone.0235984

**Published:** 2020-07-13

**Authors:** C. Alastair Grace, Martin Carr

**Affiliations:** 1 Department of Biology, University of York, Heslington, York, United Kingdom; 2 Department of Biological & Geographical Sciences, School of Applied Sciences University of Huddersfield, Huddersfield, United Kingdom; University of Helsinki, FINLAND

## Abstract

The stalk-eyed flies (Diopsidae, Diptera) are a family of approximately 100 species of calypterate dipterans, characterised by extended head capsules. Species within the family have previously been shown to possess six subfamilies of *mariner* transposons, with nucleotide substitution patterns suggesting that at least two subfamilies are currently active. The *vertumnana* subfamily has been shown to have been involved in a horizontal transfer event involving Diopsidae and a second dipteran family in the Tephritidae. Presented here are cloned and sequenced *mariner* elements from three further diopsid species, in addition to a bioinformatic analysis of *mariner* elements identified in transcriptomic and genomic data from the genus *Teleopsis*. The newly identified *mariner* elements predominantly fall into previously recognised subfamilies, however the publicly available *Teleopsis* data also revealed a novel subfamily. Three of the seven identified subfamilies are shown to have undergone horizontal transfer, two of which appear to involve diopsid donor species. One recipient group of a diopsid *mariner* is the *Bactrocera* genus of tephritid flies, the transfer of which was previously proposed in an earlier study of diopsid *mariner* elements. The second horizontal transfer, of the *mauritiana* subfamily, can be traced from the *Teleopsis* genus to the cnidarian *Hydra vulgaris*. The *mauritiana* elements are shown to be active in the recipient *H*. *vulgaris* and *transposase* expression is observed in all body tissues examined in both species. The increased diversity of diopsid *mariner* elements points to a minimum of four subfamilies being present in the ancestral genome. Both vertical inheritance and stochastic loss of TEs have subsequently occurred within the diopsid radiation. The TE complement of *H*. *vulgaris* contains at least two *mariner* subfamilies of insect origin. Despite the phylogenetic distance between donor and recipient species, both subfamilies are shown to be active and proliferating within *H*. *vulgaris*.

## Introduction

Transposable elements (TEs) are almost universal components of eukaryotic genomes [1], that are capable of driving their own replication and movement within their host genome. They are divided into two classes, based upon their transposition mechanism. The Class I elements are retrotransposons, which transpose via an RNA intermediate; in contrast, Class II elements are transposons that move as DNA molecules. Transposition of many DNA transposons is facilitated by a Transposase (Tnpase) enzyme. The Tnpase binds to the inverted terminal repeats (ITRs) that flank the transposon and generates double stranded DNA breaks in order to excise the parental element and then integrate the element into a new genomic location. Daughter elements may be generated during transposition if the double stranded break created to excise the parental element is repaired using a copy of the transposon as a template (reviewed in [2]). DNA transposons can be subdivided through the phylogenetic analysis of their Tnpase sequences. Within Metazoa, one of the most widespread forms of DNA transposon is the *Tc1*/*mariner* superfamily, named after elements originally discovered in *Caenorhabditis elegans* [3] and *Drosophila mauritiana* [4].

TEs are recognised as a major source of mutation within their hosts’ genomes. Mutations may be deleterious due to a variety of different mechanisms. Insertion mutations result from TEs integrating within or adjacent to host genes and thereby either altering their expression or mRNA sequence [5, 6], whilst recombination between similar TEs in different genomic locations can produce gross chromosomal rearrangements and result in selection against ectopic exchange [7]. The presence of TEs within their hosts’ genomes results in a metabolic burden due to RNA production, protein synthesis and the repair of double stranded DNA breaks, so the transposition process itself can be deleterious to the host organism [8]. It can be seen that for each of these processes individuals harbouring higher TE copy numbers will be at a greater selective disadvantage than those with lower copy numbers. TEs can be considered to be in a state of genomic conflict with their hosts, as their ability to proliferate may be opposed by natural selection through their hosts. Furthermore, active elements are constantly acquiring mutations and it has been proposed that TEs have a natural life cycle within their hosts, with elements entering a naïve genome and proliferating, before deactivating mutations, as well as host repression mechanisms, result in the loss of all active copies [9].

TE families may maintain on-going transposition through their horizontal transfer into a new host population. The new host is unlikely to have defences, such as RIP, RNAi or protein targeting [10–12], against the invading TE, which will only evolve once the host adapts to the new TE family [13]. Horizontal transfer has been shown to be a common feature of TE evolution [14–17] and has been shown to occur between closely related species [18], as well as species from different eukaryotic supergroups [19]. The different mechanisms which underpin horizontal transfer are currently unknown, although it has been speculated that shared parasites, viruses and introgression between closely related taxa may facilitate the transfer of TEs from one species to another [12, 20, 21]. Horizontal transfer events may be identified through incongruencies between phylogenetic trees, where TE phylogenies show strongly supported differences to host species phylogenies. As phylogenies frequently have poor support, due to the rapid rate of TE evolution [22, 23], phylogenetic trees may often be consistent with both horizontal transfer and vertical inheritance. The direction of a horizontal transfer event may however be established if TEs from one taxon are nested, with strong support, within the TEs of the second taxon. In such circumstances the nested taxon is likely to be the recipient that has acquired the TE from the donor in which its TEs are nested.

**Fig 1:**
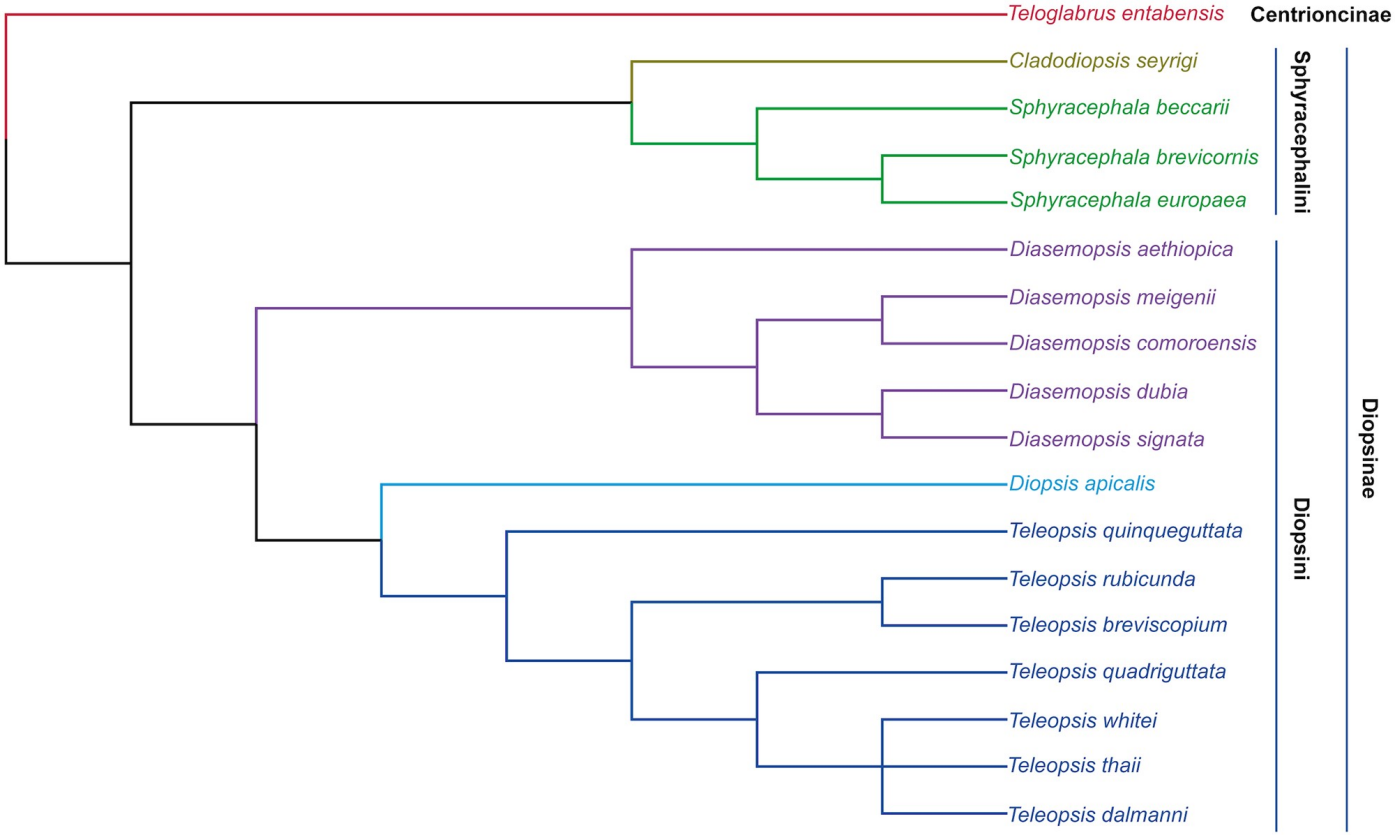
Representative phylogeny of diopsid species. Cladogram highlighting the relationships between the diopsid species involved in this study, based upon Kotrba and Balke [28] and Kotrba et al [29].

The *Tc1*/*mariner* superfamily has been extensively studied with regard to both horizontal transfer and vertical inheritance [24–26]. Carr [16] showed that 14 species of diopsid stalk-eyed fly possess a minimum of six subfamilies of the *mariner* elements, with evidence for on-going transposition uncovered in two families. Diopsids form a family of acalyptrate dipterans (Fig 1) that exhibit hypercephaly, with head capsules laterally extended into eyestalks [27]. One subfamily of the Diopsidae, Centrioncinae, is non-hypercephalic, whilst the second subfamily, Diopsinae, is only made up from hypercephalic species [28]. The Diopsinae are further divided into two tribes, the Sphyracephalini and the Diopsini [27].

One of the two identified putatively active *mariner* subfamilies within the diopsids, the *vertumnana* subfamily, presented a phylogeny consistent with the vertical inheritance of the transposon through the Diopsini tribe [16]. In addition, a *vertumnana mariner tnpase* from the dipteran *Bactrocera neohumeralis* (Tephritidae) was recovered as nested, with strong support, within the diopsid *vertumnana* sequences, consistent with the horizontal transfer of the *vertumnana* subfamily from the diopsids into *Bactrocera* [16]. The geographically close habitat ranges of *Bactrocera* and the potential donor genus *Teleopsis* led to the proposal that the horizontal transfer may have occurred in New Guinea. The species composition of *Teleopsis* is under debate [29–31], however here it is used within its broadest sense to include the putatively nested or synonymous genera *Cyrtodiopsis* and *Megalobops*.

### Experimental aims

The presented work expands upon the original diopsid *mariner* survey of Carr [16] through a PCR screen of two additional species from the Diopsinae, *Diasemopsis aethiopica* and *Diopsis apicalis*, and the first investigated species from the Centrioncinae in *Teloglabrus entabensis*. Furthermore whole genome bioinformatic surveys of *Teleopsis dalmanni* and *Sphyracephala brevicornis* were performed in order to overcome the limitations of PCR screening to investigate diopsid *mariner* diversity. Additional bioinformatic analyses were performed using RNA-Seq reads to identify novel *mariner* sequences in *Teleopsis* species and determine relative gene expression levels. The diopsid-*Bactrocera* horizontal transfer, proposed in Carr [16], is re-evaluated and two further horizontal transfers, between insects and the cnidiarian *Hydra vulgaris* (Hydridae), are analysed in depth.

## Methods

### DNA extraction and PCR

Whole flies of *D*. *aethiopica*, *Di*. *apicalis* and *Teloglabrus entabensis* were provided by Andrew Pomiankowski of University College, London. DNA was extracted from individual specimens using the Proteinase K/NaCl protocol of Carr et al. [32]. PCR was carried out in 50μl volumes (5U Abgene Red Hot DNA Polymerase, 2.5 mM MgCl2, 0.4mM dNTP), using the MAR124F and MAR276R primers of Robertson [24], which amplify multiple *mariner* subfamilies. The annealing temperature was 52°C, with an extension step of 72 °C of 1 minute per cycle other than the final cycle which utilised a 10 minute extension step. PCR was undertaken over 25 cycles.

PCR products were ligated into the pGEM-T Easy Vector (Promega) and transformed into Subcloning Efficiency DH5α Chemically Competent Cells (Invitrogen). Plasmid DNA was extracted using the Qiagen Spin Miniprep kit and *mariner* DNA sequenced using T7 and SP6 primers (Macrogen Inc, Seoul, Korea). All sequences have been deposited in GenBank (accession numbers MN719915-MN719940).

### Bioinformatic identification of novel *mariner* sequences

Genomes of *T*. *dalmanni* (assemblies NLCU01 and JXPO01), *S*. *brevicornis* (JXPL01), *H*. *vulgaris* (ACZU01 and ABRM01), *H*. *oligactis* (PJUT01) and *H*. *viridissima* (PJUU01) were downloaded from NCBI. Each was screened with RepeatMasker [33] specifying a library of *Drosophila* sequences available in RepBase [34] with the following options: *-species* drosophila *-pa* 4 *-nolow -no_is -inv -a*. Custom *mariner* RepeatMasker libraries were compiled and genomes re-screened to obtain all hits not collected using *Drosophila* sequences using the same options as above with the exception of *-lib* custom_library.

Putative miniature inverted-repeat transposable element (MITE) families were identified in the genomes of *T*. *dalmanni*, *H*. *vulgaris* and *S*. *brevicornis* using MITE Tracker [35]. The resulting families_nr.fasta file was BLASTed using *Tdmar*, *Hvmar* and *Sbmar* nucleotide queries to ascertain the regions where MITEs may have been derived from the full-length *mariner* sequences.

Full-length *mariner* elements were identified in the *T*. *dalmanni* genome by undertaking BLASTn similarity searches of the whole genome shotgun contigs using the partial, putatively autonomous, *tnpase* sequences identified through PCR and RepeatMasker screening. Contigs with *tnpase* hits for individual subfamilies were aligned with MAFFT v7.309 [36] using the L-INS-I strategy and default parameters; this strategy resulted in the *mariner* elements in the contigs being aligned with each other. Diagnostic TA target site duplications were identified to confirm that the 5’ and 3’ termini had been identified. Illumina RNA-Seq sequencing reads from *T*. *quinqueguttata* (SRX1490590, SRX1490591) and *T*. *whitei* (SRX485305) were mapped onto the complete *tnpase* (coding sequence) cds from each *T*. *dalmanni* subfamily in order to generate reconstructed species-specific sequences (S1 Dataset). Reads were mapped onto *tnpase* cds with SMALT v. 0.2.6 (https://www.sanger.ac.uk/science/tools/smalt-0). The number of reads mapped to each *tnpase* was calculated in Tablet v.1.19.09.03 [37] from the SMALT output SAM files.

A full-length *mauritiana* subfamily sequence from *H*. *vulgaris* was produced using 180bp query sequences from *Tdmar2* (NLCU01006112, 92267–90983). Reads were identified using the Trace Archive Nucleotide BLAST on the Hydra magnipapillata–wgs database. A *H*. *vulgaris mosellana* partial *tnpase* was uncovered (accession number ABRM01005801) in the BLASTn screening for the subfamily phylogeny, using *Tdmar2* as a query sequence. The ABRM01005801 hit was then used as a query sequences for the *H*. *vulgaris* whole genome shotgun contigs, using BLASTn, in order to identify contigs containing *mosellana* subfamily elements. Full-length sequences were then uncovered by aligning contigs in MAFFT. Contig ABRM01012171 was assembled with an intact *mosellana* element that possessed a premature stop codon at positions 431–433. Screening of the Trace Archive for the Hydra magnipapillata–wgs database revealed the majority of reads showed guanosine at position 431 rather than the thymine present in ABRM01012171. Replacing the thymine with guanosine results in a glutamate residue in the Tnpase, as opposed to the stop codon represented in contig ABRM01012171 (S1 Dataset).

### Phylogenetic analyses

The sequenced diopsid *mariner* clones were aligned against homologous regions of *tnpase* from publicly available diopsid sequences, as well as the uncovered *tnpase* sequences identified in the whole genome contigs of *S*. *brevicornis* and *T*. *dalmanni* and the sequence read archive (SRA) RNA-Seq files of *T*. *whitei* in MAFFT using the L-INS-I strategy and default parameters. The alignment was manually edited by eye in order to minimise indel regions. The resulting alignment was then subjected to maximum likelihood analysis with the raxmlGUI [38], using the thorough bootstrapping methodology and 1,000 bootstrap replicates. The ML tree was generated from 100 starting parsimony trees and created with the GTRCAT model, following the RAxML author’s recommendation. Bayesian inference phylogenies were created with MrBayes 3.2.6 [39] on the Cipres Science Gateway server [40]. The analyses were run with the GTR+I+Γ model and a four category gamma distribution to correct for among site rate variation. The MCMC analyses consisted of 5,000,000 generations with two parallel chain sets run at default temperatures and a sampling frequency of 1000, with a burnin value of 1250.

The diopsid *irritans* subfamily dataset was constructed only from putatively autonomous sequences generated through PCR and bioinformatic screening. Nucleotide sequences were acquired for the *mosellana* subfamily phylogeny using BLASTn, with the *tnpase* cds of *Tdmar4* used as a query sequence. The nr/nt database was screened without an organism limitation, whilst the wgs database was limited to screening Metazoa (taxid: 33208). *Bactrocera* sequences in NCBI were screened for the *vertumnana* subfamily phylogeny using the *T*. *quinqueguttata* sequence *Tqmar1*.*1* (DQ197023) with BLASTn with screened organisms limited to *Bactrocera* (taxid: 27456) in both the nr/nt and wgs databases. Extracted sequences were aligned to diopsid sequences in MAFFT. Maximum likelihood and Bayesian inference phylogenies were created for the *irritans*, *mauritiana* and *mosellana* subfamilies using the same protocols as for the diopsid *mariner* sequences.

Subfamily maximum likelihood and Bayesian inference phylogenies, created with individual insertion sequences, were generated using the same protocols as the diopsid *mariner* phylogeny. For *Hvmar1* and *Hvmar2* the Trace Archive for the Hydra magnipapillata–wgs database was screened through NCBI using the 5’ ITR and untranslated region (UTR) as query sequences. 5’ITR/UTR query sequences were also used for *Tdmar2-4* insertions phylogenies. BLASTn searches were conducted using the SRA database on the file SRX2950777, which contained 9,283,997 reads of male *T*. *dalmanni* genomic DNA. For both the *T*. *dalmanni* and *H*. *vulgaris* TEs, individual insertions were identified using the 5’ flanking DNA in the sequencing read, with reads that contained less than 12bp of flanking DNA discarded. Sequences were additionally discarded if the 5’ and 3’ termini of the ITR/UTR regions were not intact.

All alignment used in the phylogenetic analyses are presented in the Nexus format in the S2 Dataset.

### Gene expression analysis of *mariner tnpase*

Raw Illumina RNA-Seq transcriptome read files were downloaded from NCBI (see S3 Table for accession numbers, as well as the tissue type and number of reads for each file). Reads were mapped onto *tnpase* cds with SMALT. The number of reads mapped to each *tnpase* was calculated in Tablet v.1.19.09.03 [37] from the SMALT output SAM files. Normalised gene expression levels were calculated as transcripts per million (TPM) [41].

## Results

### A revised phylogeny of *mariner* within Diopsidae

Cloned PCR products amplified from genomic DNA extractions of *Diasemopsis aethiopica*, *Diopsis apicalis* and *Teloglabrus entabensis* were subject to BLASTn similarity searching, which resulted in 26 partial *mariner* sequences being identified (S1 Table). The PCR screen only identified the *vertumnana* subfamily in *Di*. *apicalis*. One clone did not show any obvious null mutations and may have been amplified from an autonomous element, whilst the other two clones contained premature stop codons in the amplified region. The *irritans*, *mauritiana* and *mellifera* subfamilies were amplified from the genomic DNA of *D*. *aethiopica*. Only one sequence, from the *irritans* subfamily, did not contain premature stop codons. The genome of the centrioncid *Te*. *entabensis* harbours a minimum of four subfamilies, with the *capitata*, *irritans*, *mellifera* and *vertumnana* subfamilies all amplified in the PCR screen. Possible autonomous elements, lacking premature stop codons, were amplified from the *irritans*, *mellifera* and *vertumnana* subfamilies, whilst the single copy amplified from the *capitata* subfamily contained multiple internal stop codons.

The newly generated sequences were combined with the diopsid *mariner* sequences from Carr [16] to produce a customised RepeatMasker [33] library, in order to screen the whole genome contigs of *T*. *dalmanni* and *S*. *brevicornis*. The *S*. *brevicornis* screen revealed the presence of two *mariner* elements with putative, albeit imperfect, ITRs (accession numbers: JXPL01000092 and JXPL01000142, S1 Dataset). Neither copy was assembled as encoding a functional *tnpase*, indicating that *mariner* is no longer active in *S*. *brevicornis*. The screen of *T*. *dalmanni* identified 93 *mariner*-like sequences within the assembled whole genome shotgun (wgs) contigs. Phylogenetic analyses of the *T*. *dalmanni* sequences resulted in four distinct clades (97–100% maximum likelihood bootstrap percentage (mlBP); 1.00 bayesian inference posterior probability (biPP)), which correspond to four different *mariner* subfamilies (S1 Fig). Confirming the results of the PCR screen in Carr [16], the *mauritiana* and *vertumnana* subfamilies were uncovered with RepeatMasker; furthermore the *irritans* subfamily and a previously unidentified subfamily were also present. The *mariner* complement of *T*. *dalmanni* recovered in the RepeatMasker screen was dominated by elements from the *mauritiana* subfamily, with 68 of 93 identified insertions belonging to this subfamily. Full length, putatively autonomous, elements were uncovered for three of the subfamilies in the *T*. *dalmanni* whole genome contigs (S1 Dataset) through the presence of inverted terminal repeats and diagnostic TA target site duplications generated by *mariner* elements [42]. No functional copy of the *vertumnana* subfamily was identified, with only degraded pseudogenes present in the assembled genome.

Further bioinformatic searching for diopsid *mariner* sequences was performed by mapping SRA sequencing reads onto the *mariner* clones sequenced here and in Carr [16], as well as the full-length *mariner tnpase* open reading frames (ORFs) identified in the contigs of *T*. *dalmanni*. RNA-Seq SRA files were screened for *T*. *quinqueguttata* and *T*. *whitei*. The *vertumnana*, *cecropia*, *mauritiana* and *mellifera* subfamilies, previously identified in *T*. *quinqueguttata* [16], were all expressed at relatively low levels (S2 Table), however no additional subfamilies were identified in the *T*. *quinqueguttata* RNA-Seq reads. From the RNA-Seq reads of *T*. *whitei* complete *tnpase* ORFs could be reconstructed for the *irritans*, *mauritiana* subfamilies as well as the novel subfamily uncovered in *T*. *dalmanni* (S1 Dataset).

**Fig 2:**
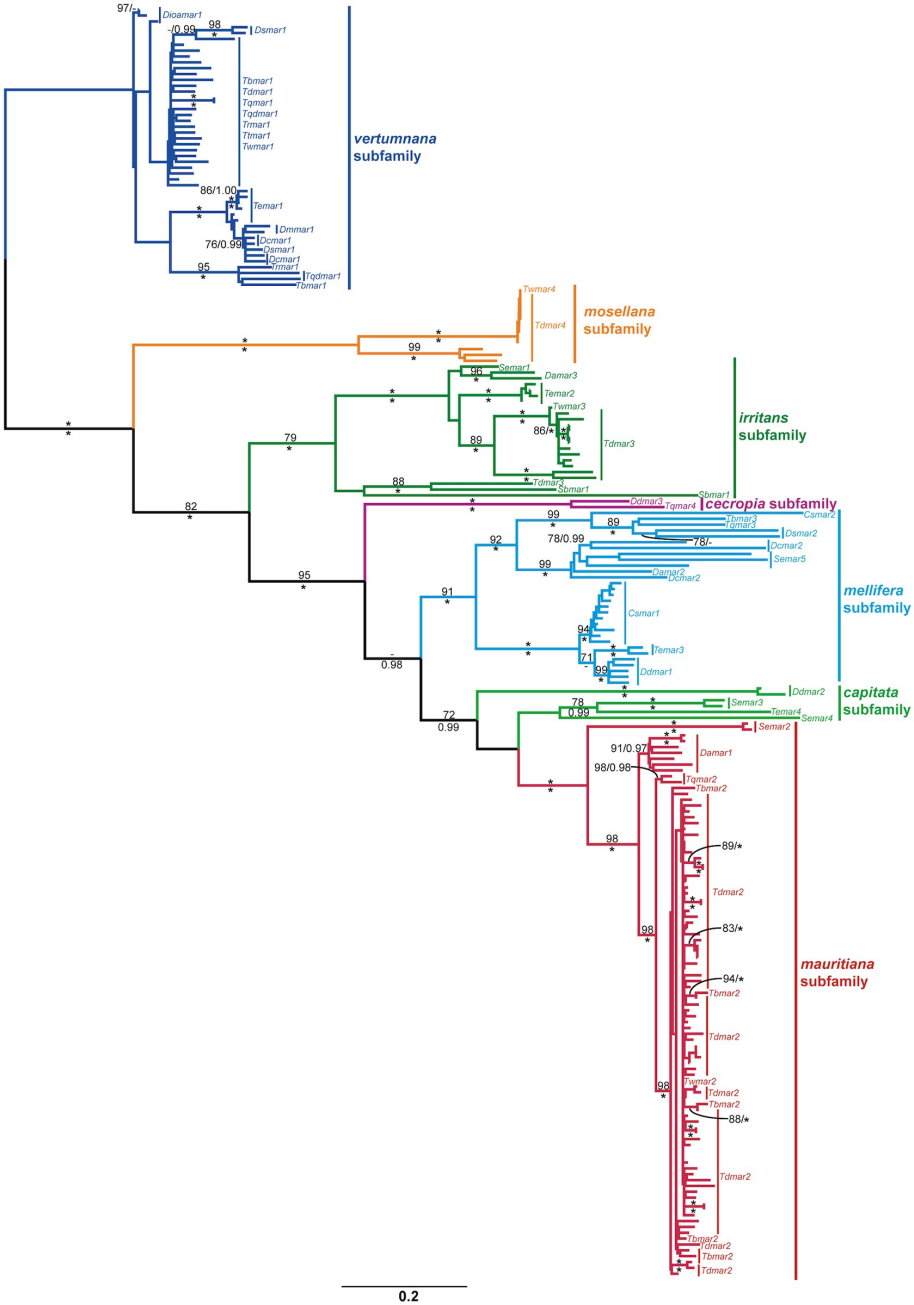
Maximum likelihood phylogeny of diopsid *mariner* sequences. The phylogeny was constructed from 480 aligned nucleotide positions using the GTRCAT model, and estimated nucleotide frequencies. Values for mlBP and biPP are shown above and below the branches respectively. 100% mlBP and 1.00 biPP are both denoted by “*”. Values <70% mlBP and <0.97 biPP are denoted by “-”. The scale bar represents the number of substitutions per site. Individual *mariner* subfamilies are bracketed and colour-coded.

The combined diopsid dataset for all identified *mariner* sequences comprised 217 sequences and was analysed using both ML and BI methodologies (Fig 2). The six *mariner* subfamilies identified in diopsids by Carr [16] were recovered, however the *capitata* subfamily was not recovered as monophyletic, albeit with only moderate phylogenetic support (55% mlBP, 0.81 biPP). A seventh subfamily was identified, with strong support (100% mlBP, 1.00 biPP), which was made up only from *mariner* elements from *T*. *dalmanni* and *T*. *whitei*. The two non-functional *S*. *brevicornis mariner* elements were recovered with strong support (79% mlBP, 1.00 biPP) as members of the *irritans* subfamily.

The presence and identity of subfamilies in the screen undertaken here and Carr [16], as well as the absence of families in the whole genome contigs of *S*. *brevicornis* and *T*. *dalmanni* are shown in Table 1.

**Table 1:** The presence of *mariner* subfamilies within screened diopsid species.

Species	*capitata*	*cecropia*	*irritans*	*mauritiana*	*mellifera*	*mosellana*	*vertumnana*
**Centrioncinae**							
*Te*. *entabensis*	*Temar4*	-	*Temar2*	-	*Temar3*	-	*Temar1*
**Sphyracephalini**							
*C*. *seyrigi*	-	-	-	-	*Csmar1*-2	-	-
*S*. *beccarii*	-	-	-	-	-	-	-
*S*. *brevicornis*	Absence^a^	Absence^a^	*Sbmar1*	Absence^a^	Absence^a^	Absence^a^	Absence^a^
*S*. *europaea*	*Semar3*, *Semar4*	-	*Semar1*	*Semar2*	*Semar5*	-	-
**Diopsini**							
*D*. *aethiopica*	-	-	*Damar3*	*Damar1*	*Damar2*	-	-
*D*. *comoroensis*	-	-	-	-	*Dcmar2*	-	*Dcmar1*
*D*. *dubia*	*Ddmar2*	*Ddmar3*	-	-	*Ddmar1*	-	-
*D*. *meigenii*	-	-	-	-	-	-	*Dmmar1*
*D*. *signata*	-	-	-	-	*Dsmar2*	-	*Dsmar1*
*Di*. *apicalis*	-	-	-	-	-	-	*Dioamar1*
*T*. *breviscopium*	-	-	-	*Tbmar2*	*Tbmar3*	-	*Tbmar1*
*T*. *dalmanni*	Absence^a^	Absence^a^	*Tdmar3*	*Tdmar2*	Absence^a^	*Tdmar4*	*Tdmar1*
*T*. *quadriguttata*	-	-	-	-	-	-	*Tqdmar1*
*T*. *quinqueguttata*	-	*Tqmar4*	-	*Tqmar2*	*Tqmar3*	-	*Tqmar1*
*T*. *rubicunda*	-	-	-	-	-	-	*Trmar1*
*T*. *thaii*	-	-	-	-	-	-	*Ttmar1*
*T*. *whitei*	-	-	*Twmar3*^b^	*Twmar2*^b^	-	*Twmar4*^b^	*Twmar1*

Dash represents the absence of the subfamily in PCR and transcriptome screens.

^a^: The subfamily is absent from the assembled whole genome contigs.

^b^: Sequences identified in RNA-Seq reads.

Due to low numbers of putatively autonomous copies, individual phylogenies were not created for the diopsid representatives of the *capitata*, *cecropia*, *mellifera* subfamilies and the novel subfamily uncovered in *Teleopsis*. The *capitata* and *mellifera* subfamilies were both identified in the centrioncid *Te*. *entabensis*, as well as the Sphyracephalini and Diopsini tribes of Diopsinae (Fig 2). The *cecropia* subfamily elements are currently limited to two species, *D*. *dubia* and *T*. *quinqueguttata*, with no additional members identified in either the PCR or bioinformatics screens conducted here.

Four subfamilies were each analysed phylogenetically. The *irritans* subfamily was identified in six species, of which five harboured potentially autonomous elements. A phylogeny of the putatively autonomous diopsid elements of the *irritans* subfamily, rooted with sequences from the centrioncid *Te*. *entabensis*, is broadly congruent with the host species phylogeny; the only element in an unexpected position is a long-branched sequence from *D*. *aethiopica* which clusters with moderate to strong support (65% mlBP; 0.98 biPP) with an *irritans tnpase* from *S*. *europaea* (S2 Fig). The phylogenies of the *mauritiana* and *vertumnana* subfamilies, as well as the novel subfamily uncovered in *T*. *dalmani* are presented individually below.

### Phylogenetic analysis of the *mosellana* subfamily

**Fig 3:**
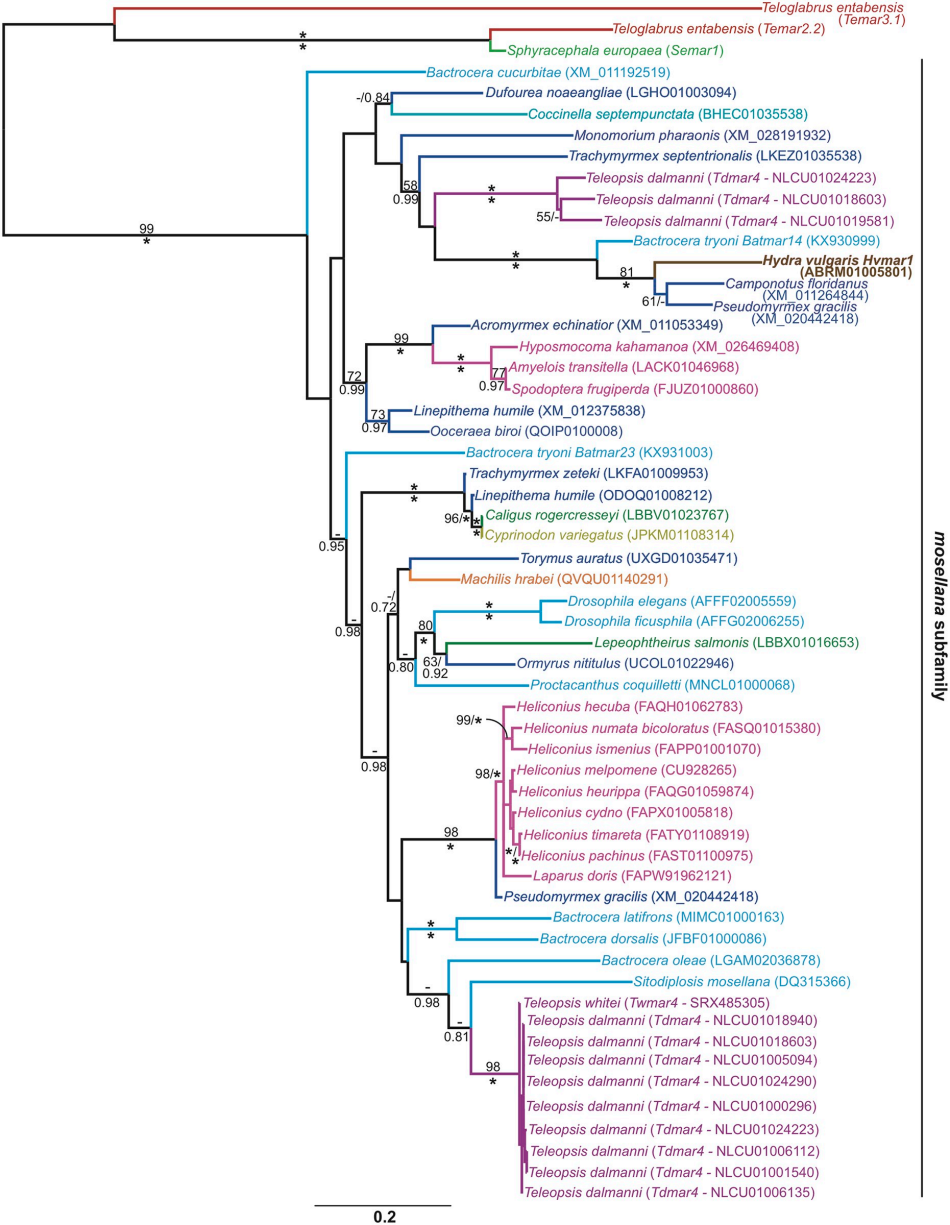
Maximum likelihood phylogeny of the *mosellana* subfamily. The phylogeny was constructed from 1032 aligned nucleotide positions using the GTRCAT model, and estimated nucleotide frequencies. The *mosellana* elements are bracketed and rooted with *tnpase* sequences from the *irritans* and *mellifera* subfamilies. Values for mlBP and biPP are shown above and below the branches respectively. Diopsid sequences are shown in purple, other dipteran sequences are shown in light blue. Dark blue sequences are from hymenopteran hosts, pink sequences from lepidopteran hosts, the orange sequence is from an archaeognath host and light green sequences are from copepod hosts. The brown sequence is from a hydrozoan host and the mustard sequence from a fish host. The outgroup sequences are from the *irritans* subfamily (*Semar1* and *Temar2*.*2*) and the *mellifera* subfamily (*Temar3*.*1*). 100% mlBP and 1.00 biPP are both denoted by “*”. Values <50% mlBP and <0.70 biPP are denoted by “-”. The scale bar represents the number of substitutions per site.

The seventh *mariner* subfamily, identified within the whole genome shotgun contigs of *T*. *dalmanni* and RNA-Seq transcriptome reads of *T*. *whitei*, was not uncovered in the diopsid PCR screen of Carr [16]. In order to investigate the evolutionary origins of the subfamily in the diopsids BLASTn similarity searching, through NCBI, was undertaken with the nucleotide sequence of a putatively autonomous *tnpase* from *T*. *dalmanni*. Only the top hit uncovered for each species was extracted for phylogenetic analysis. The screen uncovered the presence of 41 *tnpase* sequences that clustered with the novel *Teleopsis* sequences (95% mlBP; 1.00 biPP, Fig 3). The earliest published sequence from the subfamily was identified in the genome of the dipteran *Sitodiplosis mosellana* and accordingly the subfamily is named here as the *mosellana* subfamily.

The *Teleopsis* sequences fell within two distinct groups in the *mosellana* subfamily, however there were no strongly supported branches (≥70%mlBP and ≥0.97biPP) separating the two clusters (Fig 3). Of the two groups, one consisted of three non-autonomous *T*. *dalmanni* sequences which all harboured premature stop codons. The second group possessed sequences from both *T*. *dalmanni* and *T*. *whitei*, with both species harbouring putatively functional *tnpase* sequences. The potentially active *Teleopsis* elements formed a monophyletic group with *tnpase* sequences from three *Bactrocera* species and *S*. *mosellana*, however this clade of dipteran elements had no phylogenetic support.

Thirty nine of the *mosellana* sequences were uncovered from pancrustacean species, with hosts falling within the insect orders Archaeognatha, Coleoptera, Diptera, Hymenoptera and Lepidoptera, as well as the crustacean Copepoda order. The two remaining sequences were identified in the whole genome shotgun contigs of the hydrozoan cnidarian *Hydra vulgaris* (synonymised with *H*. *magnipapillata*, *H*. *littoralis* and *H*. *attenuata*) and the ray-finned fish *Cyprinodon variegatus*. The sequence from the *C*. *variegatus* contig showed a single nucleotide difference (99.9% nucleotide identity) from a *mosellana* subfamily element uncovered in the copepod *Caligus rogercresseyi*. This is consistent with a horizontal transfer between the two species, with *C*. *variegatus* being a marine fish and *C*. *rogercresseyi* a sea louse parasite of fish. The more biologically plausible direction of transfer would be from *C*. *rogercresseyi* to *C*. *variegatus*, as the fish *mariner* sequence is nested within those of the pancrustaceans by three strongly supported branches (all ≥96% mlBP and 1.00 biPP). An alternative explanation is that the sequenced genomic DNA of *C*. *variegatus* was contaminated by DNA from *C*. *rogercresseyi*. Consistent with this hypothesis only a single copy of the transposon was uncovered in the whole genome contigs of *C*. *varietgatus*, present in a contig (JPKM01108314) which only contained 53bp of flanking DNA. Two copies, possessing different flanking DNA, of the *mosellana* element were identified in *C*. *rogercresseyi* contigs (LBBV01023767 and LBBU01012271). Mapping RNA-Seq reads of *C*. *rogercresseyi* (54.6 million reads, SRX864101-2 and SRX1481244) to the *mosellana* subfamily sequence (accession number LBBV01023767) revealed 468 reads that spanned the entire coding region. The subfamily however could not be identified in *C*. *variegatus* RNA-Seq reads (124.7 million reads, SRX3140005-6, SRX3140009-11, SRX5103143, SRX5103155 and SRX5103167). The lack of expression in *C*. *variegatus* suggests either an unsuccessful horizontal transfer or contamination of genomic DNA during whole genome sequencing.

A full-length consensus sequence, labelled *Hvmar1*, was generated for the *mosellana* subfamily element in the genome of *H*. *vulgaris* (S1 Dataset). The *H*. *vulgaris tnpase* clustered with *mariner* elements from the dipteran *Bactrocera tryoni* and the hymenopteran ants *Camponotus floridanus* and *Pseudomyrmex gracilis* (100% mlBP, 1.00biPP, Fig 3). *Hvmar1* exhibited 83.7% and 87.0% nucleotide identity with the *C*. *floridanus* (accession number XM_011264844) and *P*. *gracilis* (accession number XM_020442418) *mariner* elements respectively, despite cnidarians and insects last sharing a common ancestor approximately between 600–700 million years ago [43–45]. *Hvmar1* appears to be a genuine component of the *H*. *vulgaris* genome, as similarity searching of the NCBI Hydra magnipapillata wgs Trace Archive with BLASTn revealed 148 distinct 5’ termini. Furthermore, mapping RNA-Seq reads to the *tnpase* also showed *Hvmar1* to be expressed in whole polyps, as well as head, foot, tentacle and body tissue (S2 Table).

The high nucleotide identity between *Hvmar1* and the hymenopteran *mariners*, as well as the nested position of *Hvmar1* within the insect *mosellana* sequences is consistent with a horizontal transfer event. The donor species would appear to be an insect, although the donor insect order cannot be confirmed, as *Hvmar1* clusters with *mariner* elements from dipteran and hymenopteran species but is not nested within either group. Similarity searching of whole genome contigs, with BLASTn, using *Hvmar1* as a query sequence failed to uncover *mosellana* sequences in either *H*. *viridis* or *H*. *oligactis*, as well as the genomes of other cnidarians. The absence of the subfamily within other *Hydra* species is consistent with *H*. *vulgaris* being the recipient species of the horizontal transfer event in Cnidaria.

### A reassessment of the diopsid-*Bactrocera vertumnana* horizontal transfer

Carr [16] proposed a putative horizontal transfer event between Diopsini stalk-eyed flies and the tephritid *Bactrocera neohumeralis*. Due to the *B*. *neohumeralis* sequence (clone Bnmar29, accession number AF348438.1) being nested within the *Teleopsis* sequences, it was suggested that the direction of transfer was from *Teleopsis* to *Bactrocera*, although the actual donor and recipient species could not be confirmed. The identification of *vertumnana* sequences from two additional diopsid species (*Te*. *entabensis* and *Di*. *apicalis*), as well as the additional sequencing of whole genomes from *Bactrocera* species, allowed a review of the phylogenetic relationships between *vertumnana* elements from diopsid and *Bactrocera* hosts.

**Fig 4:**
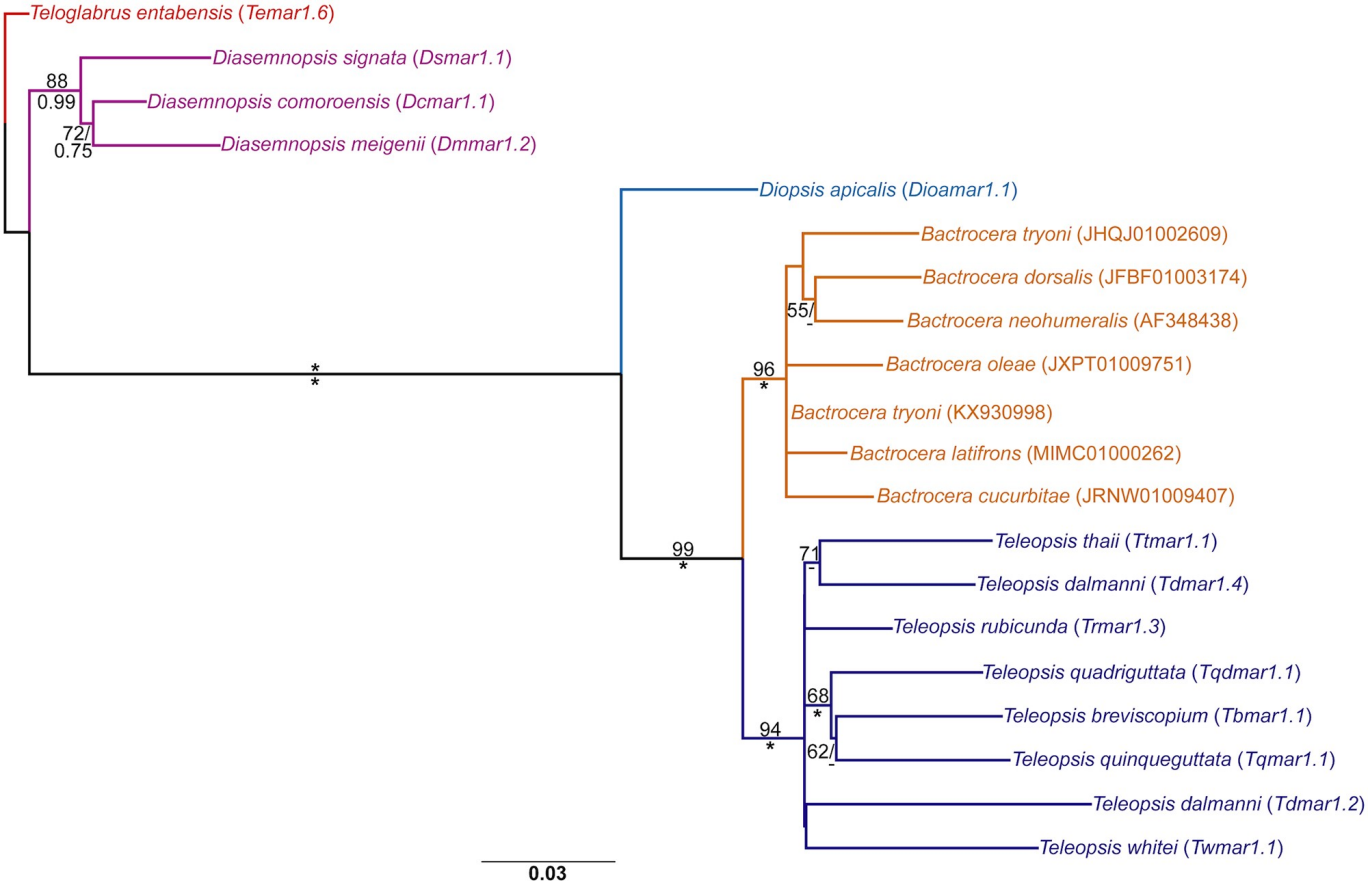
Maximum likelihood phylogeny of the *vertumnana* subfamily. The phylogeny was constructed from 510 aligned nucleotide positions using the GTRCAT model and estimated nucleotide frequencies. Support values are shown in the same format as Fig 3.

BLASTn similarity searching of *Bactrocera* wgs contigs and the nr/nt database, using *Tqmar1*.*1* (Accession Number DQ197023), uncovered *vertumnana* sequences from five species in addition to clone Bnmar29 from *B*. *neohumeralis*. The *vertumnana* subfamily therefore has a greater distribution within *Bactrocera* than recognised in Carr [16]. Diopsid *vertumnana* sequences, taken from all twelve species in which the subfamily has been identified, were aligned with seven *Bactrocera* elements. A single sequence was used for each diopsid species in which the subfamily has been identified with the exception of *T*. *dalmanni*; due to internal deletions two sequences were used for *T*. *dalmanni*. The resulting alignment was subjected to maximum likelihood and Bayesian inference analyses (Fig 4). The *Bactrocera* elements formed a strongly supported monophyletic group (96% mlBP, 1.00 biPP), as did the *vertumnana* sequences from *Teleopsis* (94% mlBP, 1.00 biPP) and *Diasemopsis* (88% mlBP, 0.99 biPP). The expanded *vertumnana* phylogeny therefore rejects the placement of the *B*. *neohumeralis* transposon within the grouping of *Teleopsis* elements recovered in the Carr [16] ML phylogeny. Rooting the phylogeny with the *mariner* sequenced from the earliest branching diopsid genus, *Teloglabrus*, recovers the expected relationships between elements from the host diopsid genera under the model of vertical inheritance. The *Bactrocera* elements are recovered as nested within the Diopsini *vertumnana* elements (100% mlBP, 1.00 biPP), as the sister-group to the *tnpase*s sequenced from *Teleopsis* species (99% mlBP, 1.00 biPP). Re-rooting the phylogeny with the *Bactrocera* sequences fails to recover any of the expected genera relationships based upon the host species phylogeny. All further potential rooting of the phylogeny also fail to recover the expected genera relationships based upon the host species phylogeny. The most biologically plausible rooting for the phylogeny is therefore between the *Teloglabrus tnpase* and the remaining transposons, with the tephritid *vertumnana* being recovered as the sister-group to the *Teleopsis* transposons. The increased dataset presented here therefore provides further evidence for a horizontal transfer event of the *vertumnana* subfamily from the Diopsini to *Bactrocera*.

### Phylogenetic evidence for a cross-phyla horizontal transfer event from *Teleopsis* to *Hydra*

BLAST screening of the newly sequenced *mariner* elements from *D*. *aethiopica* unexpectedly recovered a high scoring hit (E value: 5e-175) from *H*. *vulgaris* (accession number U51187, clone Hydra.vulgaris.6) when *Damar1*.*1* of the *mauritiana* subfamily was used as a query sequence. The only other *mariner* sequences uncovered with similarly high BLAST scores were from other diopsid species. A BLASTn screen of the *H*. *vulgaris* whole-genome shotgun contigs, using sequence Hydra.vulgaris.6 as a query sequence, confirmed the presence of 218 similar sequences recovered with E values of 0.0 indicating that the original Hydra.vulgaris.6 sequence was a genuine component of the *H*. *vulgaris* genome and not a PCR contaminant. Robertson [46] highlighted the relationship of clone Hydra.vulgaris.6 with insect *mariner* sequences, but did not propose whether a horizontal transfer donor was a cnidarian or an insect.

The finding raised the possibility of the identification of a second horizontal transfer event of a *mariner* subfamily from diopsids, as well as a second insect to *Hydra* horizontal transfer. A reciprocal BLASTn of Hydra.vulgaris.6 against diopsid sequences in the nr/nt database resulted in high scoring hits (E value: 0.0, query coverage: 100%, nucleotide identity: >95%) for *mariner* sequences deposited from *T*. *breviscopium*, *T*. *dalmanni* and *T*. *quinqueguttata*. A BLASTn screen of the whole-genome shotgun contigs of *T*. *dalmanni* uncovered a complete, intact *mauritiana mariner* element (Accession Number NLCU01006112, position 92,267–90,983), designated as *Tdmar2* on the basis of identity with the sequenced clones generated by Carr [16]. *Tdmar2* possessed 27bp inverted terminal repeats (ITRs) and a putative *tnpase* ORF of 1,038bp in length (S1 Dataset).

Screening the NCBI Sequencing Trace Archive of the *H*. *vulgaris* genome with 180bp query sequences of *Tdmar2* uncovered hits across the entire element. The concatenated hits produced a putative *mauritiana* subfamily element, designated as *Hvmar2*, which exhibited 31 nucleotide differences from *Tdmar2* out of 1,287 sites (97.6% nucleotide identity). The coding regions of *Tdmar2* and *Hvmar2* showed 19 nucleotide differences (98.2% nucleotide identity), which resulted in 14 amino acid differences between the putative Tnpases.

**Fig 5:**
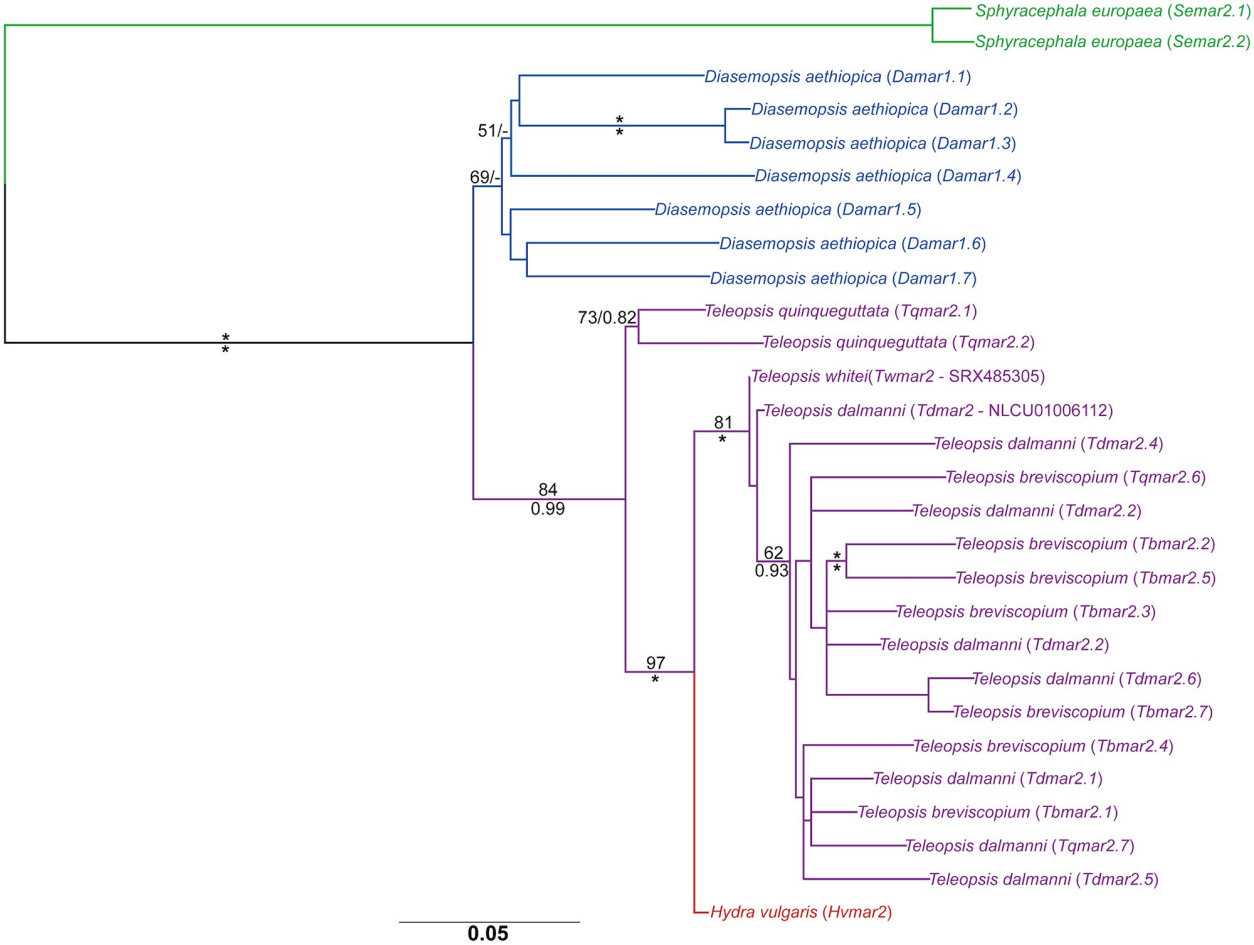
Maximum likelihood phylogeny of the *mauritiana* subfamily. The phylogeny was constructed from 492 aligned nucleotide positions using the GTRCAT model and estimated nucleotide frequencies. Support values are shown in the same format as Fig 3.

Similarity screening with BLASTn of the wgs contigs of *H*. *oligactis* and *H*. *viridis*, as well as all available cnidarian whole-genome contigs in NCBI failed to uncover orthologous *mauritiana* subfamily sequences. Phylogenetic analyses of *Hvmar2* and the diopsid *mauritiana* elements clustered the *Hvmar2* with the *Teleopsis* elements in a group with robust support (84% mlBP, 0.99 biPP, Fig 5) and furthermore nested *Hvmar2* within the *Teleopsis* sequences (97% mlBP, 1.00 biPP). Rooting the *mauritiana* sequences with those from the Sphyracephalini *S*. *europaea* recovered the expected relationships between the diopsid TEs based upon their host species, consistent with their vertical inheritance since the origin of the Diopsinae. An alternative rooting, between *Hvmar2* and the diopsid elements, failed to recover the expected species relationships, indicating that this is not the correct root for the phylogenetic tree. The *mauritiana* subfamily *Hvmar2* subfamily therefore appears to have had a recent origin from within the diopsids and specifically from the *Teleopsis* genus. *T*. *dalmanni* does not appear to be the donor species for *Hvmar2*, as *Tdmar2* is recovered as being more closely related to *mauritiana* elements from both *T*. *whitei* and *T*. *breviscopium* in Fig 5. Based upon the phylogeny the donor species would appear to be a closer relative of *T*. *breviscopium*, *T*. *dalmanni* and *T*. *whitei* than *T*. *quinqueguttata*, as the *T*. *quinqueguttata* elements are recovered with strong support at the base of the *Teleopsis mauritiana* elements. The basal position of the *T*. *quinqueguttata* elements mirrors the basal position of *T*. *quinqueguttata* in the host species genus [28, 29], further highlighting the reliability of the phylogenetic signal in the *mariner* sequences.

### *Mauritiana tnpase* is expressed in the genomes of *Teleopsis* species and *H*. *vulgaris*

The phylogenetic analyses of *tnpase* from the *mauritiana* subfamily indicated that an active *mariner* element had undergone horizontal transfer from an unknown *Teleopsis* species into the cnidarian *H*. *vulgaris*. In order to investigate this proposed transfer the activity of *mariner* was analysed in both *Teleopsis*, as the putative donor genus, and *H*. *vulgaris*, from the putative recipient genus.

RNA-Seq reads were mapped onto the *mariner* subfamily *tnpase* sequences of *T*. *dalmanni*, *T*. *quinqueguttata* and *T*. *whitei* (S2 Table). Within *T*. *dalmanni* it was possible to investigate gene expression within the male and female germ cells, as well as whole larvae and adult head cells. Consistent with the lack of identified autonomous *vertumana* elements in *T*. *dalmanni*, the subfamily exhibited the lowest mapping coverage (<1.5 TPM, ≤0.001% of RNA-Seq reads in all five tissue types), albeit based upon a shorter CDS (S2 Table). The *irritans*, *mauritiana* and *mellifera* subfamilies were all expressed in both ovary and testes cells. Expression was also observed in male and female heads, raising the possibility that transposition occurs in both somatic and germline cells. Across the examined five tissue types in *T*. *dalmanni*, *tnpase* TPM and the absolute number of mapped reads was higher for the *mosellana* subfamily (*Tdmar4*) than the *mauritiana* subfamily (*Tdmar2*). *T*. *whitei* testes expression patterns were similar to those of *T*. *dalmanni*, with the *vertumnana* TPM being an order of magnitude lower than values of the *irritans*, *mauritiana* and *mosellana* subfamilies. As in *T*. *dalmanni* testes, the *mosellana* subfamily TPM was more than sevenfold than higher than the value of the *mauritiana* subfamily. In contrast to *T*. *dalmanni* and *T*. *whitei*, the *vertumnana* subfamily showed the highest TPM in *T*. *quinqueguttata* testes. However only 39 out of 84,490,068 sequencing reads mapped to the *cecropia*, *mauritiana* and *mellifera* subfamilies, indicating that the other subfamilies may either be non-functional or silenced in *T*. *quinqueguttata* testes.

Unlike the diopsids, *H*. *vulgaris* lacks a sequestered germline [47], as gametes develop from interstitial cells which also produce neurons, cnidocytes and secretory cells [48, 49]. As with *Hvmar1* of the *mosellana* subfamily, *Hvmar2* was shown to be expressed in all cell types (S2 Table), consistent with its on-going transposition. Mapping coverage for the *mosellana* subfamily was fourfold to eightfold higher compared to the *mauritiana* subfamily for all examined tissues, mirroring the expression patterns observed in *T*. *dalmanni* and *T*. *whitei*.

### Phylogenetic analyses of individual *mariner* insertions in the genomes of *H*. *vulgaris* and *T*. *dalmanni*

The expression of *tnpase* is required for the transposition of *mariner* in both species. However, the observed expression does not confirm that the *mauritiana* elements are transposing, since the expression of *tnpase* mRNA could reflect the presence of a repressor isoform, such as the KP protein that supresses transposition of the *P* element in *Drosophila melanogaster* [8, 50]. The *mariner* elements in both *H*. *vulgaris* and *T*. *dalmanni* were therefore subjected to phylogenetic analysis in order to uncover evidence of on-going transposition. Upon transposition daughter elements should possess identical sequences to their parental elements in phylogenetic trees, despite being present at different genomic locations. Nucleotide differences will accumulate over time, as the parental and daughter elements begin to diverge following transposition.

A phylogeny of the *Hvmar1* 5’ ITR/UTR region revealed that insertions were mainly present on short terminal branches (S3 Fig), with 62 identical copies at different genomic locations. The *Hvmar2* phylogeny of 103 insertions was also generated from 5’ ITR/UTR sequences. The phylogeny was similar to that of *Hvmar1*, with a large number of short branched sequences, as well as 26 identical paralogous sequences. At the base of the phylogeny was a weakly supported grouping of long branched sequences, which appear to be older elements that are no longer transposing. The long branch sequences contained unique indels, consistent with their greater antiquity in the *H*. *vulgaris* genome, whilst the presence of identical paralogous sequences indicate that there is on-going transposition of both *Hvmar1* and *Hvmar2* in *H*. *vulgaris*.

The 5’ termini sequences identified in the sequencing reads of *T*. *dalmanni* also uncovered identical paralogous copies for the subfamilies *Tdmar2-4* (S4 Fig). Within the 86 sequences of *Tdmar2*, four insertions at different genomic locations showed identical sequences; for *Tdmar3* there were 9 identical paralogous insertions across 134 sequences. In contrast to the RepeatMasker *tnpase* screen of the whole genome contigs, which only uncovered 11 *Tdmar4* sequences, the BLAST screen of the 5’ ITR/UTR of sequencing reads identified 2,422 distinct insertions. Of those, 1,790 copies were identical to other copies in different genomic locations, indicating a high level of recent transposition of the *mosellana* subfamily in *T*. *dalmanni*.

### *Mariner*-derived MITEs are abundant in the genome of *T*. *dalmanni*

Due to the discrepancy between the results of the *T*. *dalmanni* RepeatMasker *tnpase* screen, where *Tdmar2* appeared to have the highest copy number, and the ITR/UTR wgs Trace Archive BLAST screen, which was dominated by *Tdmar4* insertions, the genomes of *T*. *dalmanni*, *S*. *brevicornis* and *H*. *vulgaris* were screened for MITEs. MITEs are non-autonomous transposons that possess ITR sequences, but lack internal coding *tnpase* sequences [51]. MITE families are derived from autonomous transposons and are mobilised via the Tnpase enzymes of autonomous element copies [52]. The *T*. *dalmanni* screen uncovered the presence of 342 putative MITE families, of which 55 appeared to have originated from *mariner* elements (Table 2). Within the *mariner*-derived MITEs 25 families had an origin within *Tdmar4* of the *mosellana* subfamily and *Tdmar4*-derived MITEs contributed 2017 out of 2489 MITE insertions in the whole genome contigs (Table 3). Consistent with the lack of autonomous *Tdmar1* copies, the *vertumnana* subfamily possessed the fewest MITEs and only contributed 73 MITE insertions, split across eight families.

**Table 2:** MITE sequences BLASTed against full-length sequences *Hvmar1-2*, *Sbmar1* and *Tdmar1-4*.

Species	Total number of MITE families	Number potentially *mariner* -derived
**Diopsidae**		
*S*. *brevicornis*	36	0
*T*. *dalmanni*	342	55
**Hydridae**		
*H*. *vulgaris*	126	0

**Table 3:** Characterisation of MITE families for each *mariner* subfamily in *T*. *dalmanni*.

Subfamily	No. of MITE families	Total copy number
*Tdmar1*	8	73
*Tdmar2*	5	98
*Tdmar3*	17	301
*Tdmar4*	25	2017

MITEs sharing identity with multiple subfamilies were assigned to that with the most significant e-value.

The *S*. *brevicornis* genome possessed 36 MITE families, none of which appeared to have a *mariner*-derived origin (Table 2), further highlighting the paucity of *mariner*-like elements within the species. In contrast to *S*. *brevicornis* the genome of *H*. *vulgaris* was rich in MITE elements. A total of 126 MITE families were identified, however none of the families were found to have an origin from *mariner* elements (Table 2).

## Discussion

### Multiple *mariner* subfamilies were present in the ancestral diopsid

The original diopsid *mariner* study of Carr [16] identified six subfamilies of *mariner* transposons within 14 species of diopsid, however, with the exception of the *vertumnana* subfamily, no attempt was made to determine the evolutionary origins of the subfamilies. The sequencing of thirteen *mariner* clones from the centrioncid *Te*. *entabensis*, allows greater insight into the subfamilies present in the last common ancestor (LCA) of diopsids.

Through the use of subfamily phylogenies, as well as the distribution of subfamilies across species, it is possible to estimate the latest evolutionary points of origin of the subfamilies within the diopsids (S5 Fig). The *capitata*, *irritans*, *mellifera* and *vertumnana* subfamilies are all present in the genome of the centrioncid *Te*. *entabensis* as well as multiple Diopsinae species, consistent with their presence in the diopsid LCA. Subfamily phylogenies of both *irritans* and *vertumnana* also indicate their vertical inheritance since the origin of the Diopsidae.

The *mauritiana* subfamily was amplified in species across the Diopsinae, but not from the centrioncid *Te*. *entabensis*. Only 13 clones were sequenced from the genomic DNA of *Te*. *entabensis*, so it is possible that the absence of the *mauritiana* subfamily was due to the limited sample size. The distribution of *mauritiana* elements, as well as the phylogenetic relationships presented in Fig 5 indicate that the subfamily was present in the genome of the Diopsinae LCA, but an earlier origin in the ancestor of all diopsids cannot be excluded on the basis of the current dataset.

The *cecropia* subfamily has only been identified within Diopsini species (Table 1), but its absence in both the Sphyracephalini and Centrioncinae is currently mainly posited on small-scale PCR screens which may not uncover low copy number, or non-functional degenerate, subfamilies. Finally, only two *Teleopsis* species have been shown to harbour *mosellana* elements, consistent with a late origin of the subfamily in the diopsids. The subfamily has not been amplified through PCR in any diopsid species or the earlier screen of *H*. *vulgaris* genomic DNA [46]. The WVPHEL amino acid motif on which the Robertson [24] forward degenerate *mariner* primer was designed is not present in the reconstructed *mosellana* Tnpase sequences in *T*. *dalmanni*, *T*. *whitei* and *H*. *vulgaris* (S1 Dataset). Conceptual translations of the *mosellana tnpase* sequences used to generate the subfamily phylogeny in Fig 3 also lack the WVPHEL motif. The homologous region of the Tnpase could be translated in 33 species and 19 encoded the amino acids LVPKEL. The Robertson primers appear to lack specificity to *mosellana* elements in a broad range of species; therefore the absence of amplified PCR product may be the result of failed primer binding rather than the absence of *mosellana* elements in a species’ genome. The loss of the highly conserved WVPHEL motif will require the design of alternative degenerate primers to amplify *mosellana* elements from genomic DNA. The lack of the *mosellana* subfamily from the transcriptome of *T*. *quinqueguttata* is consistent with an origin of the subfamily in *Teleopsis* after the lineage leading to *T dalmanni* and *T*. *whitei* split from the *T*. *quinqueguttata* lineage. It remains however possible that the *mosellana* subfamily has greater antiquity in *Teleopsis*, and perhaps other diopsid taxa, and has undergone stochastic loss in *T*. *quinqueguttata*.

The *mariner* subfamilies present in the *T*. *dalmanni* genome have persisted for sufficient time in order to generate non-autonomous MITE families. The MITE complement is dominated by transposons generated from *Tdmar4* of the *mosellana* subfamily, however all four subfamilies have produced multiple MITE families. The available data suggest that the ancestral diopsid possessed a diverse complement of *mariner* elements, with a minimum of four subfamilies residing in the genome. Due to the limitations of small-scale PCR screens and limited whole genome availability across the Diopsidae, the origins of the *cecropia*, *mauritiana* and *mosellana* subfamilies are unresolved and they may be either ancestral or more recent acquisitions into diopsid genomes. Stochastic loss of *mariner* subfamilies has occurred within the diopsids, as can been seen by the absence of any active copies in the sequenced genome of *S*. *brevicornis*, as well as the absence of *mariner* elements in PCR screen of the genomic DNA of *S*. *beccarii* reported in Carr [16]. *S brevicornis* is a closer relative of *S*. *europaea*, a species which possess multiple *mariner* subfamilies, than *S*. *beccarrii* (Fig 1), indicating the absence of *mariner* elements in *S*. *beccarrii* and *S*. *brevicornis* is due to independent losses.

Within *T*. *dalmanni* the *irritans*, *mauritiana* and *mosellana* subfamilies are all expressed in both the male and female germline. This finding is consistent with *mariner* transposition occurring in both sexes, unlike the sex-restricted transposition of some TE families, such as *copia* and *Doc*, observed in *D*. *melanogaster* [53].

### The *vertumnana* subfamily diopsid-*Bactrocera* horizontal transfer event

The phylogeny presented here recovers a nested position of the *Bactrocera* elements within those of the Diopsini in the *vertumana* subfamily (Fig 4). Carr [16] proposed a horizontal transfer event within New Guinea from either a *Teleopsis* species, or related diopsid, to *Bactrocera*, based primarily upon the phylogeny of the *mariner* elements, but also the then recognised distribution of *Teleopsis* and *Bactrocera* species. More recently, Feijen and Feijen [54] stated that the direction of horizontal transfer should be re-evaluated, as they considered *Teleopsis* species to be absent from New Guinea. The enlarged *vertumnana* phylogeny presented here, with additional diopsid and *Bactrocera mariner* sequences, provides an ideal opportunity to reassess the inheritance of the subfamily. The increased diversity of *Bactrocera mariner* elements from the *vertumnana* subfamily highlights that the proposed horizontal transfer event did not occur into *B*. *neohumeralis*, but within an ancestor of at least five *Bactrocera* species. The monophyly of the *Bactrocera* elements, which are nested within the paraphyletic Diopsini *vertumnana* elements with strong support, points toward the diopsids being the donor group and *Bactrocera* being the recipients. Feijen and Feijen’s [54] alternative argument failed to take into account the required horizontal transfer of a *vertumnana mariner* from the Australasian *B*. *neohumeralis* into African *Diasemopsis* species if the hypothesis of *Bactrocera* being the donor group was correct. The revised phylogeny presented here provides additional evidence for the Diopsidae being the donor to *Bactrocera*. The putatively recipient *Bactrocera* species are present across South Asia [55] and not confined to Australasia, thereby expunging the argument that the direction of transfer could not have been from diopsids to *Bactrocera* due to the absence of *Teleopsis* from New Guinea. The presence of the *vertumana* subfamily in additional African diopsid genera, in *Diopsis* and *Teloglabrus*, would require a further two independent, intercontinental horizontal transfer events, under the *Bactrocera* to Diopsidae horizontal transfer route. The phylogeny presented here requires a single horizontal transfer event from Diopsini to *Bactrocera* within South East Asia, as was the case in the original Carr [16] phylogenetic tree. However the alternative route from *Bactrocera* to Diopsidae requires a minimum of four independent horizontal transfer events, into the genera *Diasemopsis*, *Diopsis*, *Teleopsis* and *Teloglabrus*.

### Horizontal transfer events of *mariner* from insects to *H*. *vulgaris*

The genome of *H*. *vulgaris* is rich in TEs, with approximately 57% of the genome being made up from over 500 TE families [49]. The *H*. *vulgaris* genome is considerably larger than that of its distant congener *H*. *viridis* [56] and the difference has been speculated to be the result of bursts of transposition by multiple TE families [49, 56, 57]. DNA transposons contribute to 21% of the *H*. *vulgaris* genome, with *mariner* elements alone making up 4% of the sequenced genome [49]. PCR screens of the genomes of *H*. *vulgaris*, including the North American subspecies/sister-species *H*. *littoralis*, have identified *mariner* elements from the *capitata*, *cecropia*, *irritans* and *mauritiana* subfamilies [46].

Two of the *mariner* subfamilies identified here in diopsid species are also present in the genome of *H*. *vulgaris*, these being the *mauritiana* and *mosellana* subfamilies. Orthologous *mariner* elements appear to be absent from the sequenced genomes of both *H*. *oligactis* and *H*. *viridis*, consistent with their invasion of *Hydra* occurring after the *H*. *vulgaris* lineage split from other *Hydra* approximately 21–28 million years ago [58]. The presence of the subfamilies in the ancestors of either all three *Hydra* species or only *H*. *vulgaris* and *H*. *oligactis* is a less parsimonious explanation, which would require multiple stochastic loss events in addition to the gains of the two subfamilies through horizontal transfer. The *mosellana* subfamily, designated *Hvmar1*, was not identified in a previously published PCR screen in either *H*. *vulgaris* or *H*. *littoralis* genomic DNA [46], however this may be due to the lack of subfamily primer specificity due to the loss of the WVPHEL amino acid motif. The phylogeny of the *mosellana* subfamily presented here indicates a horizontal transfer event from an unknown insect donor into *H*. *vulgaris* (Fig 3). The lack of a clear donor species, or even donor insect order, has resulted in *Hvmar1* being placed on a relatively long branch within the subfamily phylogeny; it is therefore unclear as to whether the horizontal transfer was an ancient or more recent event. The transfer of *Hvmar1* appears to have been a successful one. The *tnpase* is expressed across multiple cell types and, based upon the ITR/UTR phylogeny which showed 62 identical paralogous copies, *Hvmar1* is currently transposing within the *H*. *vulgaris* genome.

In contrast to the *mosellana* subfamily, the *mauritiana* subfamily, designated here as *Hvmar2*, was amplified by Robertson with clone Hydra.vulgaris.6 [46]. The relationship of Hydra.vulgaris.6 to other *mariner* elements was not robustly resolved in the Robertson phylogeny, but it was nested within a clade of insect *mariner* elements. No diopsid *mariner* elements were included in the phylogeny, with Hydra.vulgaris.6 clustering with *mauritiana* elements from *D*. *mauritiana* and the hymenopteran *Myrmecia occidentalis*. The *mauritiana* phylogeny presented here robustly nests *Hvmar2* within the diopsid elements and highlights a putative horizontal transfer between an unknown *Teleopsis* species and *H*. *vulgaris*. The Tnpases of the *mauritiana* subfamily were shown by Carr [16] to be evolving under purifying selection on their amino acid sequences, indicating the elements are active and therefore potentially viable donors. The *mauritiana* horizontal transfer into *H*. *vulgaris* has also been successful, with *Hvmar2 tnpase* expression observed across body tissues and multiple identical paralogous insertions identified in the whole genome sequencing reads.

The mechanism, or mechanisms, that have facilitated the horizontal transfer events from insects into *H*. *vulgaris* are difficult to envisage. Aquatic cnidarians and terrestrial insects sharing mutual parasites or viruses appears to be unlikely, given their approximately 600 million year divergence time and different habitats. Terrestrial insect larvae or imagos which fall into the water column may be preyed upon by *Hydra*, which are known to feed upon dipteran larvae and can engulf prey items in excess of 30mm in length [59]. As *Teleopsis* species, as well as members of other diopsid genera, often live over bodies of water [29, 30, 60] opportunistic predation may potentially allow diopsid *mariner* DNA to be taken up by *Hydra* cells resulting in horizontal transfer.

The lack of *mariner*-derived MITEs is consistent with both *Hvmar1* and *Hvmar2* being recent invaders in the *H*. *vulgaris* genome and contrasts with the proliferation of MITE families in *T*. *dalmanni*. The absence of orthologous families of *Hvmar1* and *Hvmar2* in the whole genome contigs of both *H*. *viridis* and *H*. *oligactis* suggests that the horizontal transfer events from insect donors occurred within the *H*. *vulgaris* species complex. The lack of available sequence data means that the approximate age of the *mosellana* transfer cannot be gauged, however the very low nucleotide divergence (~2.5%) between *Tdmar2* and *Hvmar2* suggest that the *mauritiana* transfer occurred very recently in the evolutionary history of *Hydra*. A donor *Teleopsis* species has not been identified, with the *Hvmar2 tnpase* showing 98.2% and 98.5% nucleotide identity to the *tnpase* sequences of *Tdmar2* and *Twmar2* respectively. *Teleopsis* species are restricted to eastern Asia, with many species present in south east Asia [30, 54] therefore a broader screen of *Teleopsis* species will be required in order to determine if the genus harbours the donor species. The subspecies, or strain, of *H*. *vulgaris* which has been shown to harbour *Hvmar2* in its genome, *H*. *magnipapillata*, was isolated from Japan and closely related populations are present in south east Asia [58], overlapping with *Teleopsis* species and indicating a possible east Asian location for the *mauritiana* subfamily horizontal transfer event. The *H*. *vulgari*s AEP strain is a North American laboratory-produced line, generated through a cross of strains from California and Pennsylvania [58]. The presence of *Hvmar2* in the transcriptome reads from the AEP strain highlights the transcontinental movement of this *mauritiana* element in the *H*. *vulgaris* global population.

## Conclusions

Sequencing of *mariner* elements from the basal centrioncid *Te*. *entabensis* points to a minimum of four subfamilies being present in the ancestral diopsid. A total of seven subfamilies have now been identified within Diopsidae genomes. The identification of the *mosellana* subfamily in whole genome sequence data highlights the inherent dangers of relying upon degenerate primers in PCR screening for *mariner* elements, as the widely used primers designed by Robertson [37] do not amplify this subfamily. Two diopsid *mariner* subfamilies appear to have undergone horizontal transfer to species outside of the family. One of the putative recipient species, *H*. *vulgaris*, has also acquired a *mariner* element from a second, unidentified, insect donor. Despite the great evolutionary distance between insects and cnidarians, both transferred *mariner* elements have successfully proliferated in *Hydra* contributing to the diverse TE complement of this species.

## Supporting information

S1 Dataset Annotated sequences of the *mariner* subfamilies characterized in *S*. *brevicornis*, *Teleopsis* species and *Hydra vulgaris*. The full-length sequences for each identified subfamily are presented, along with putative open-reading frames, untranslated regions and flanking repeats. Conceptual translations of encoded proteins are provided.
(TXT)

S2 Dataset Alignments used in the phylogenetic analyses. Alignments are provided in the Newick format. Columns present within square brackets were excluded from the phylogenetic analyses.
(TXT)

S1 Fig Maximum likelihood phylogeny of the *T*. *dalmanni mariner* sequences uncovered in whole genome shotgun contigs using the customized *mariner* RepeatMasker library. The phylogeny was constructed from 1099 aligned nucleotide positions using the GTRCAT model, and estimated nucleotide frequencies. Values for mlBP and biPP are shown above and below the branches respectively. 100% mlBP and 1.00 biPP are both denoted by “*”. Values <50% mlBP and <0.70 biPP are denoted by “-”. The scale bar represents the number of substitutions per site. Individual *mariner* subfamilies are bracketed and colour-coded.
(PDF)

S2 Fig Maximum likelihood phylogeny of diopsid *irritans tnpase* sequences. The phylogeny was constructed from 500 aligned nucleotide positions using the GTRCAT model, and estimated nucleotide frequencies. The phylogeny layout is the same as in S1 Fig.
(PDF)

S3 Fig Maximum likelihood phylogenies of *mariner* 5’ ITR/UTR sequences within the *H*. *vulgaris* genome. Phylogenies were generated using the GTRCAT model with empirical base frequencies. A) *Hvmar1* created from 246 aligned nucleotide positions, B) *Hvmar2* created from 291 aligned nucleotide positions. OTU labels are the 5’ flanking DNA of the ITR. The phylogeny layouts are otherwise the same as in S1 Fig.
(PDF)

S4 Fig Maximum likelihood phylogenies of *mariner* 5’ ITR/UTR sequences within the *T*. *dalmanni* genome. Phylogenies were generated using the GTRCAT model with empirical base frequencies. A) *Tdmar2* created from 187 aligned nucleotide positions, B) *Tdmar3* created from 204 aligned nucleotide positions, C) *Tdmar4* created from 197 aligned nucleotide positions. OTU labels are the 5’ flanking DNA of the ITR for A and B. C is presented as a radial tree and both the support values and OTU labels are omitted due to the large number of sequences. The phylogeny layouts are otherwise the same as in S1 Fig.
(PDF)

S5 Fig Representative diopsid phylogeny showing the latest possible points of origins of *mariner* subfamilies. Circles represent the putative origin points of the subfamilies. The tree layout is the same as Fig 1.
(PDF)

S1 Table *mariner* sequences generated in this study. (DOCX)

S2 Table Number of RNA-Seq reads mapped to *mariner tnpase* sequences. (DOCX)

S3 Table SRA RNA-Seq files used in gene expression analyses. (DOCX)

## References

[1] Pritham EJ. Transposable elements and factors influencing their success in eukaryotes. J Hered. 2009;100: 648–655. 10.1093/jhered/esp065 19666747 PMC2877548

[2] Hartl DL. Discovery of the transposable element *Mariner*. Genetics 2001;157: 471–476. 11156971 10.1093/genetics/157.2.471 PMC1461507

[3] Emmons SW, Yesner L, Ruan K-s, Katzenberg D. Evidence for a transposon in *Caenorhabditis elegans*. Cell 1983;32: 55–65. 10.1016/0092-8674(83)90496-8 6297788

[4] Jacobson JW, Medhora MM, Hartl DL. Molecular structure of a somatically instable transposable element in *Drosophila*. Proc Natl Acad Sci USA 1986;83: 8684–8688. 10.1073/pnas.83.22.8684 3022302 PMC386995

[5] Charlesworth B, Charlesworth D. The population dynamics of transposable elements. Genet Res 1983;42: 1–27.

[6] Montgomery EA, Charlesworth B, Langley CH. A test for the role of natural selection in the stabilization of transposable element copy number in a population of *Drosophila melanogaster*. Genet Res 1987;49: 31–41. 10.1017/s0016672300026707 3032743

[7] Langley CH, Montgomery EA, Hudson R, Kaplan N, Charlesworth B. On the role of unequal exchange in the containment of transposable element copy number. Genet Res 1988;52: 223–235. 10.1017/s0016672300027695 2854088

[8] Brookfield JFY. Models of transposition repression in P-M hybrid dysgenesis and by zygotically encoded repressor proteins. Genetics 1991;128: 471–486. 1649073 10.1093/genetics/128.2.471 PMC1204483

[9] Brookfield JFY. The ecology of the genome–mobile DNA elements and their hosts. Nat Rev Genet 2005;6: 128–36. 10.1038/nrg1524 15640810

[10] Tabara H, Sarkissian M, Kelly WG, Fleenor J, Grishok A, Timmons L, et al The *rde*-1 gene, RNA interference and transposon silencing in *C*. *elegans*. Cell 1999;99: 123–132. 10.1016/s0092-8674(00)81644-x 10535731

[11] Hood ME, Katawczik M, Giraud T. Repeat-induced point mutation and the population structure of transposable elements in *Microbotryum violaceum*. Genetics 2005;170: 1081–1089. 10.1534/genetics.105.042564 15911572 PMC1451165

[12] deHaro D, Kines KJ, Sokolowski M, Dauchy RT, Streva VA, Hill SM, et al Regulation of L1 expression and retrotransposition by melatonin and its receptor: implications for cancer risk associated with light exposure at night. Nucleic Acids Res 2014;42: 7694–7707. 10.1093/nar/gku503 24914052 PMC4081101

[13] Kofler R. Dynamics of transposable element invasions with piRNA clusters. Mol Biol Evol 2019;36: 1457–1472. 10.1093/molbev/msz079 30968135 PMC6573471

[14] Daniels SB, Peterson KR, Strausbaugh LD, Kidwell MG, Chovnick A. Evidence for horizontal transmission of the *P* transposable element between *Drosophila* species. Genetics 1990;124: 339–355. 2155157 10.1093/genetics/124.2.339 PMC1203926

[15] Sánchez-Gracia A, Maside X, Charlesworth B. High rate of horizontal transfer of transposable elements in *Drosophila*. Trends Genet 2005;21: 200–203. 10.1016/j.tig.2005.02.001 15797612

[16] Carr M. Multiple subfamilies of *mariner* transposable elements are present in stalk-eyed flies (Diptera: Diopsidae). Genetica 2008;132: 113–122. 10.1007/s10709-007-9157-2 17562187

[17] Kuraku S, Qiu H, Meyer A. Horizontal transfers of Tc1 elements between teleost fishes and their vertebrate parasites, lampreys. Genome Biol Evol 2012;4: 929–936. 10.1093/gbe/evs069 22887124 PMC3516227

[18] Carr M, Bensasson D, Bergman CM. Evolutionary genomics of transposable elements in *Saccharomyces cerevisiae*. PLoS One. 2012;7: e50978 10.1371/journal.pone.0050978 23226439 PMC3511429

[19] Southworth J, Grace CA, Marron AO, Fatima N, Carr M. A genomic survey of transposable elements in the choanoflagellate *Salpingoeca rosetta* reveals selection on codon usage. Mobile DNA-UK 2019;10: 44. 10.1186/s13100-019-0189-9 PMC6875170 31788034

[20] Yoshiyama M, Tu Z, Kainoh Y, Honda H, Shono T, Kimura K. Possible horizontal transfer of a transposable element from host to parasitoid. Mol Biol Evol 2001;18: 1952–1958. 10.1093/oxfordjournals.molbev.a003735 11557800

[21] Gilbert C, Chateigner A, Ernenwein L, Barbe V, Bézier A, Herniou EA, et al Population genomic supports baculoviruses as vectors of horizontal transfer of insect transposons. Nat Comm 2014;5: 3348. 10.1038/ncomms4348 PMC3948050 24556639

[22] Peterson-Burch BD, Voytas DF. Genes of the Pseudoviridae (*Ty1/copia* Retrotransposons). Mol Biol Evol 2002;19: 1832–1845. 10.1093/oxfordjournals.molbev.a004008 12411593

[23] Carr M, Nelson M, Leadbeater BSC, Baldauf SL. Three families of LTR retrotransposon are present in the genome of the choanoflagellate *Monosiga brevicollis*. Protist 2008;159: 579–590. 10.1016/j.protis.2008.05.001 18621583

[24] Robertson HM. The *mariner* element is widespread in insects. Nature 1993;362: 241–245. 10.1038/362241a0 8384700

[25] Robertson HM, Lampe DJ. Recent horizontal transfer of a *mariner* transposable element among and between Diptera and Neuroptera. Mol Biol Evol 1995;12: 850–862. 10.1093/oxfordjournals.molbev.a040262 7476131

[26] Casse N, Bui QT, Nicolas V, Renault S, Bigot Y, Laulier M. Species sympatry and horizontal transfers of *mariner* transposons in marine crustacean genomes. Mol Phylogenet Evol 2006;40: 609–619. 10.1016/j.ympev.2006.02.005 16690328

[27] Shillito JF. The genera of Diopsidae (Insecta: Diptera) Zool J Linn Soc 1971;50: 287–295.

[28] Kotrba M, Balke M. The systematic position of *Cladodiopsis* Séguy, 1949 and the origin of sexual dimorphism in stalk-eyed flies (Diptera: Diopsidae) inferred from DNA sequence data. Mol Phyl Evol 2006;38: 843–847. 10.1016/j.ympev.2005.11.009 16406820

[29] Kotrba M, Carr M, Balke M. The systematic position of *Diopsina* Curran, 1928 (Diptera: Diopsidae) inferred from DNA sequence data. Insect Syst Evol 2010;41: 295–302.

[30] Földvári M, Pomiankowski A, Cotton S, Carr M. A comprehensive morphological and molecular description of a new *Teleopsis* species (Diptera, Diopsidae) from Thailand. Zootaxa 2007;1620: 37–51

[31] Feijen HR, Feijen C. An annotated catalogue of the stalk-eyed flies (Diopsidae: Diptera) of India with description of new species in *Megalabops* Frey and *Teleopsis* Rondani. Israel J Entom 2019;49: 35–72.

[32] Carr M, Cotton S, Földvári M, Kotrba M. A description of a new species of *Diasemopsis* (Diptera, Diopsidae) from the Comoro Islands with morphological, molecular and allometric data. Zootaxa 2006;1211: 1–19.

[33] Smit, AFA, Hubley, Green P (2013–2015) RepeatMasker Open-4.0.

[34] Bao W, Kojima KK, Kohany O. Repbase Update, a database of repetitive elements in eukaryotic genomes. Mobile DNA 2015;6: 11 10.1186/s13100-015-0041-9 26045719 PMC4455052

[35] Crescente JM, Zavallo D, Helguera M, Vanzetti LS. MITE Tracker: an accurate approach to identify miniature inverted-repeat transposable elements in large genomes. BMC Bioinformatics 2018;19: 348 10.1186/s12859-018-2376-y 30285604 PMC6171319

[36] Katoh K, Standley DM. MAFFT multiple sequence alignment software version 7: improvements in performance and usability. Mol Biol Evol 2013;30: 772–780. 10.1093/molbev/mst010 23329690 PMC3603318

[37] Milne I, Stephen G, Bayer M, Cock PJA, Pritchard L, Cardle L, et al Using Tablet for visual exploration of second-generation sequencing data. Brief Bioinform 2013;14: 193–202. 10.1093/bib/bbs012 22445902

[38] Silvestro D, Michalak I. raxmlGUI: a graphical front-end for RAxML. Org Divers Evol. 2011;12: 335–337

[39] Ronquist F, Teslenko M, van der Mark P, Ayres DL, Darling A, Hohna S, et al MrBayes 3.2: efficient Bayesian phylogenetic inference and model choice across a large model space. Syst Biol. 2012;61: 539–42. 10.1093/sysbio/sys029 22357727 PMC3329765

[40] Miller MA, Pfeiffer W, Schwartz T. Creating the CIPRES Science Gateway for inference of large phylogenetic trees. In: Proceedings of the Gateway Computing Environments Workshop (GCE), 14 Nov. 2010, New Orleans, LA, pp. 1–8.

[41] Wagner GP, Kin K, Lynch VJ. Measurement of mRNA abundance using RNA-seq data: RPKM measure is inconsistent among samples. Theory Biosci 2012;131: 281–285. 10.1007/s12064-012-0162-3 22872506

[42] Plasterk RHA, Izsvák Z, Ivics Z. Resident aliens: The *Tc1*/*mariner* superfamily of transposable elements. Trends Genet 1999;15: 326–332. 10.1016/s0168-9525(99)01777-1 10431195

[43] dos Reis M, Thawornwattana Y, Angelis K, Telford MJ, Donoghue PC, Yang Z. Uncertainty in the timing of origin of animals and the limits of precision in molecular timescales. Curr Biol 2015;25: 2939–2950. 10.1016/j.cub.2015.09.066 26603774 PMC4651906

[44] Miyazawa H, Ueda C, Yahata K, Su ZH. Molecular phylogeny of Myriapoda provides insights into evolutionary patterns of the mode in post-embryonic development. Sci Rep 2014;4: 4127 10.1038/srep04127 24535281 PMC3927213

[45] Peterson KJ, Cotton JA, Gehling JG, Pisani D. The Ediacaran emergence of bilaterians: congruence between the genetic and the geological fossil records. Philos Trans R Soc Lond B Biol Sci 2008;363: 1435–1443. 10.1098/rstb.2007.2233 18192191 PMC2614224

[46] Robertson HM. Multiple *mariner* transposons in flatworms and Hydras are related to those of insects. J Hered 1997;88: 195–201. 10.1093/oxfordjournals.jhered.a023088 9183847

[47] Bosch TCG, David CN. Stem cells of *Hydra magnigpapillata* can differentiate into somatic and germ line cells. Dev Biol 1987;121: 182–191.

[48] Martínez DE. Mortality patterns suggest lack of senescence in Hydra. Exp Gerontology 1998;33: 217–225. 10.1016/s0531-5565(97)00113-7 9615920

[49] Chapman JA, Kirkness EF, Simakov O, Hampson SE, Mitros T et al The dynamic genome of *Hydra*. Nature 2010;464: 592–596. 10.1038/nature08830 20228792 PMC4479502

[50] Lee CC, Mul YM, Rio DC. The *Drosophila* P-element KP repressor protein dimerizes and interacts with multiple sites on P-element DNA. Mo Cell Biol 1996;16: 5616–5622. 10.1128/mcb.16.10.5616 PMC231561 8816474

[51] Wessler SR, Bureau TE, White SE. LTR-retrotransposons and MITEs: important players in the evolution of plant genomes. Curr Opin Genet Dev 1995;5: 814–821. 10.1016/0959-437x(95)80016-x 8745082

[52] Feschotte C, Osterlund MT, Peeler R, Wessler SR. DNA-binding specificity of rice *mariner*-like transposases and interactions with *Stowaway* MITEs. Nucleic Acids Res 2005;44: 2153–2165. 10.1093/nar/gki509 PMC1079968 15831788

[53] Pasyukova EG, Nuzhdin SV, Filatov DA. The relationship between the rate of transposition and transposable element copy number for *copia* and *Doc* retrotransposons of *Drosophila melanogaster*. Genet Res 1998;72: 1–11. 10.1017/s0016672398003358 9802257

[54] Feijen HR, Feijen C. On the biogeographic range of the genus *Teleopsis* Rondani (Diptera: Diopsidae), with redescription of *Teleopsis sykesii* from India and description of a new species from Borneo. Zool Med Leident 2011;85: 141–159.

[55] Qin Y, Paini DR, Wang C, Fang Y, Li Z. Global establishment risk of economically important fruit fly species (Tephritidae). PLoS ONE 2015;10: e0116424 10.1371/journal.pone.0116424 25588025 PMC4294639

[56] Zacharias H, Anokhin B, Khalturin K, Bosch TCG. Genome sizes and chromosomes in the basal metazoan *Hydra*. Zoology 2004;107: 219–227. 10.1016/j.zool.2004.04.005 16351940

[57] Wong WY, Simakov O, Bridge DM, Cartwright P, Bellantuono AJ, Kuhn A, et al Expansion of a single transposable element family is associated with genome-size increase and radiation in the genus *Hydra*. Proc Natl Acad Sci USA 2019;116: 22915–22917. 10.1073/pnas.1910106116 31659034 PMC6859323

[58] Martínez DE, Iñiguez AR, Percell KM, Willner JB, Signorovitch J, Campbell RD. Phylogeny and biogeography *of Hydra* (Cnidaria: Hydridae) using mitochondrial and nuclear DNA sequences. Mol Phylogenet Evol 2010;57: 403–410. 10.1016/j.ympev.2010.06.016 20601008

[59] Deserti MI, Esquius KS, Escalante AH, Acuña FH. Trophic ecology and diet of *Hydra vulgaris* (Cnidaria; Hydrozoa). Anim Biol 2017;67: 287–300.

[60] Feijen HR, Feijen C. *Diopsis* (Diopsidae, Diptera) with unusual wing spots: two new species from Malawi with a longer eye span in females than in males. Zool Med Leiden 2009;83: 701–722.

