# Supplementary figures and images for: The evolutionary history of mariner elements in stalk-eyed flies reveals the horizontal transfer of transposons from insects into the genome of the cnidarian Hydra vulgaris
Source: PLoS One. 2020 Jul 13;15(7):e0235984. doi: 10.1371/journal.pone.0235984 (PMC7357744; doi:10.1371/journal.pone.0235984)

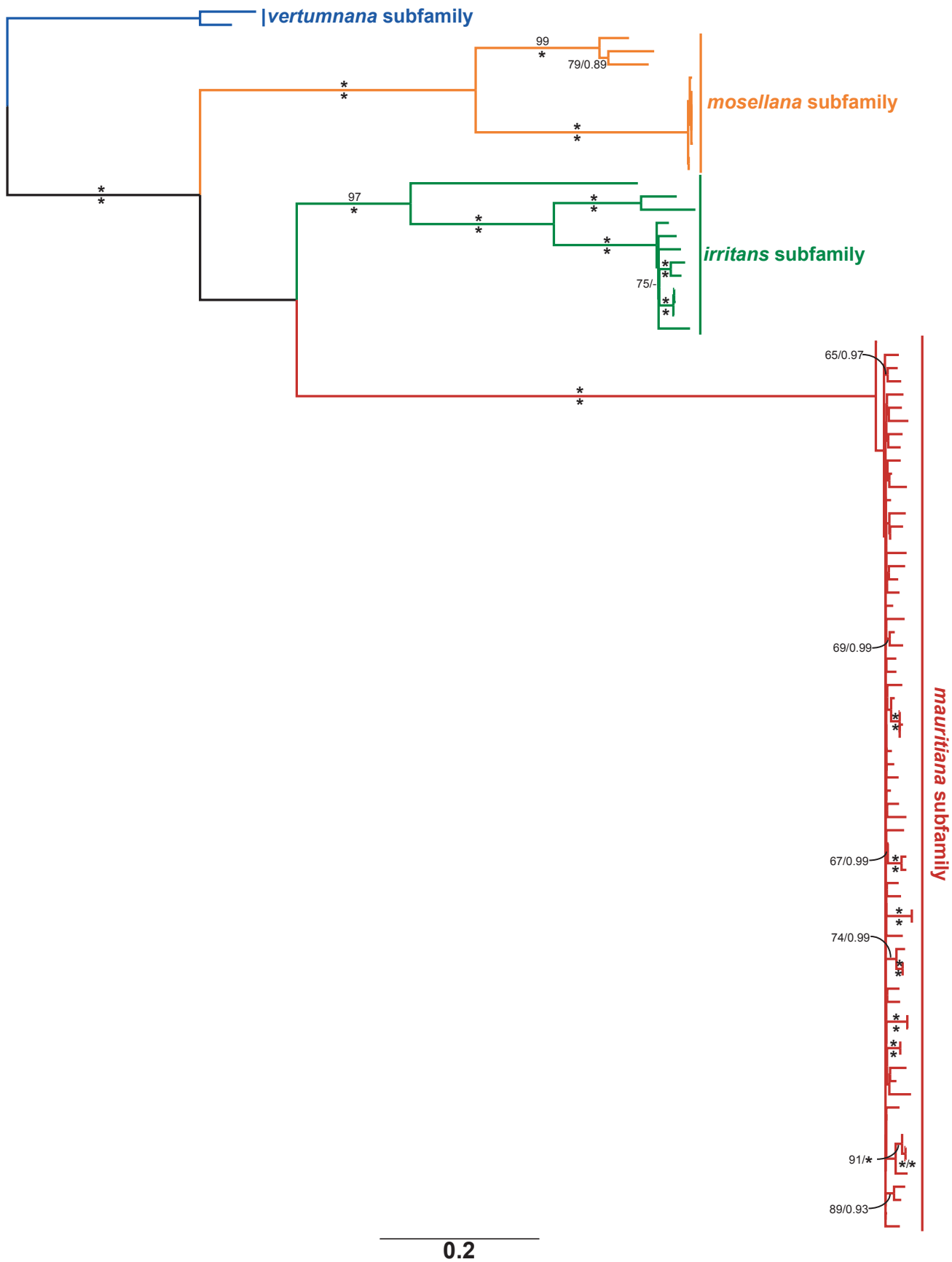

Supplement: S1 Fig — The phylogeny was constructed from 1099 aligned nucleotide positions using the GTRCAT model, and estimated nucleotide frequencies. Values for mlBP and biPP are shown above and below the branches respectively. 100% mlBP and 1.00 biPP are both denoted by “*”. Values <50% mlBP and <0.70 biPP are denoted by “-”. The scale bar represents the number of substitutions per site. Individual mariner subfamilies are bracketed and colour-coded. (PDF) [file pone.0235984.s003.pdf]

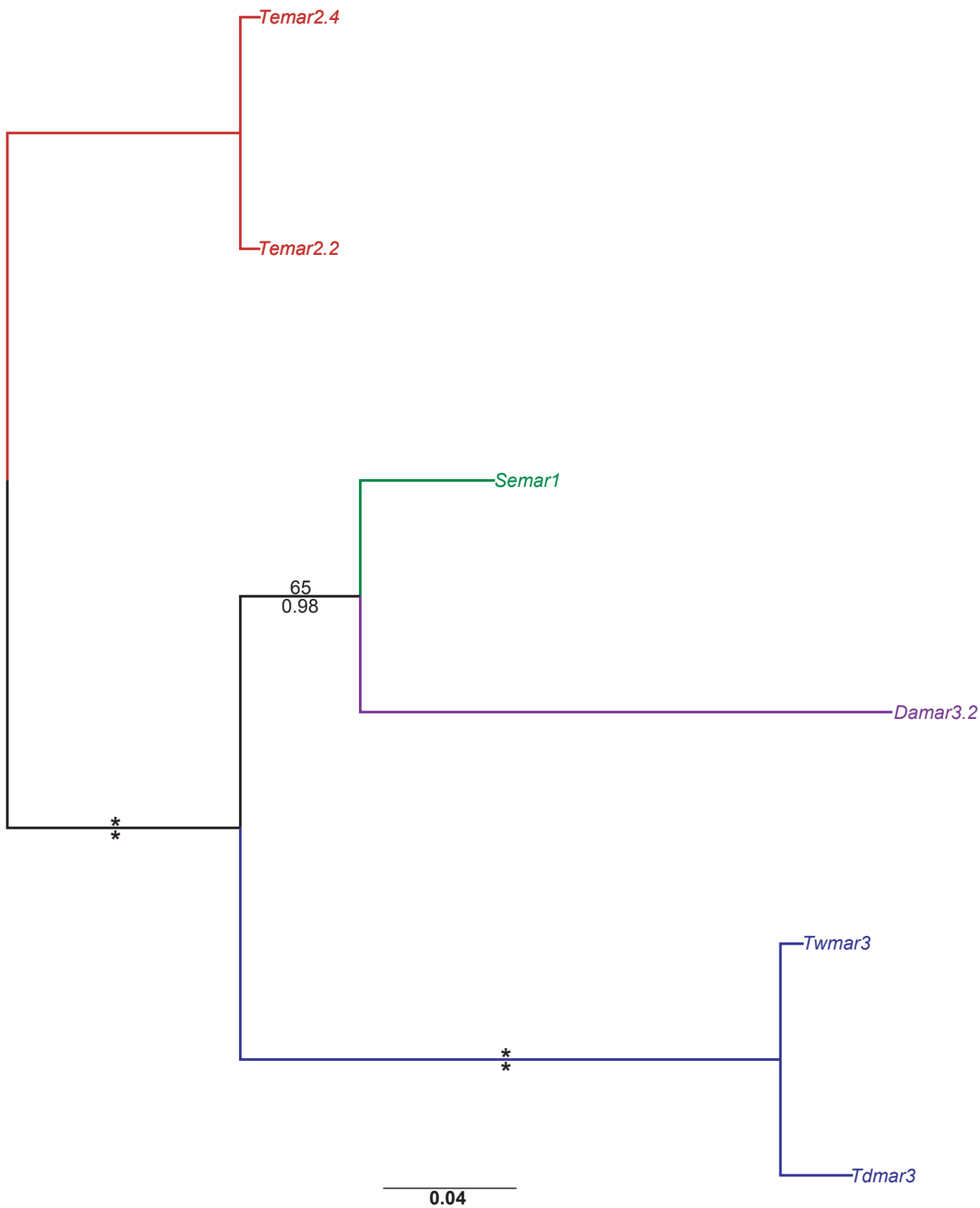

Supplement: S2 Fig — The phylogeny was constructed from 500 aligned nucleotide positions using the GTRCAT model, and estimated nucleotide frequencies. The phylogeny layout is the same as in S1 Fig. (PDF) [file pone.0235984.s004.pdf]

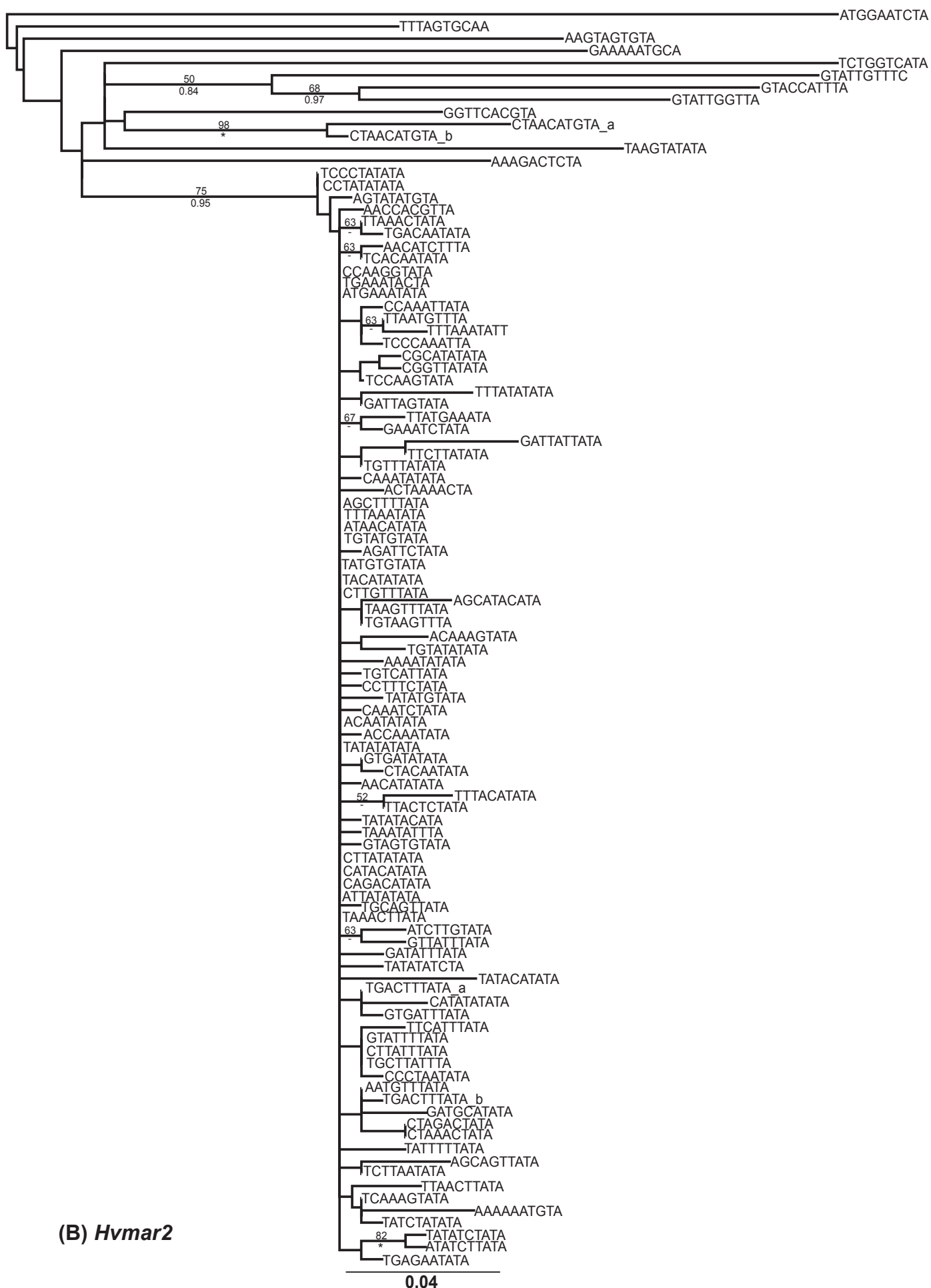

(B) *Hvmr2*

Supplement: S3 Fig — Phylogenies were generated using the GTRCAT model with empirical base frequencies. A) Hvmar1 created from 246 aligned nucleotide positions, B) Hvmar2 created from 291 aligned nucleotide positions. OTU labels are the 5’ flanking DNA of the ITR. The phylogeny layouts are otherwise the same as in S1 Fig. (PDF) [file pone.0235984.s005.pdf]

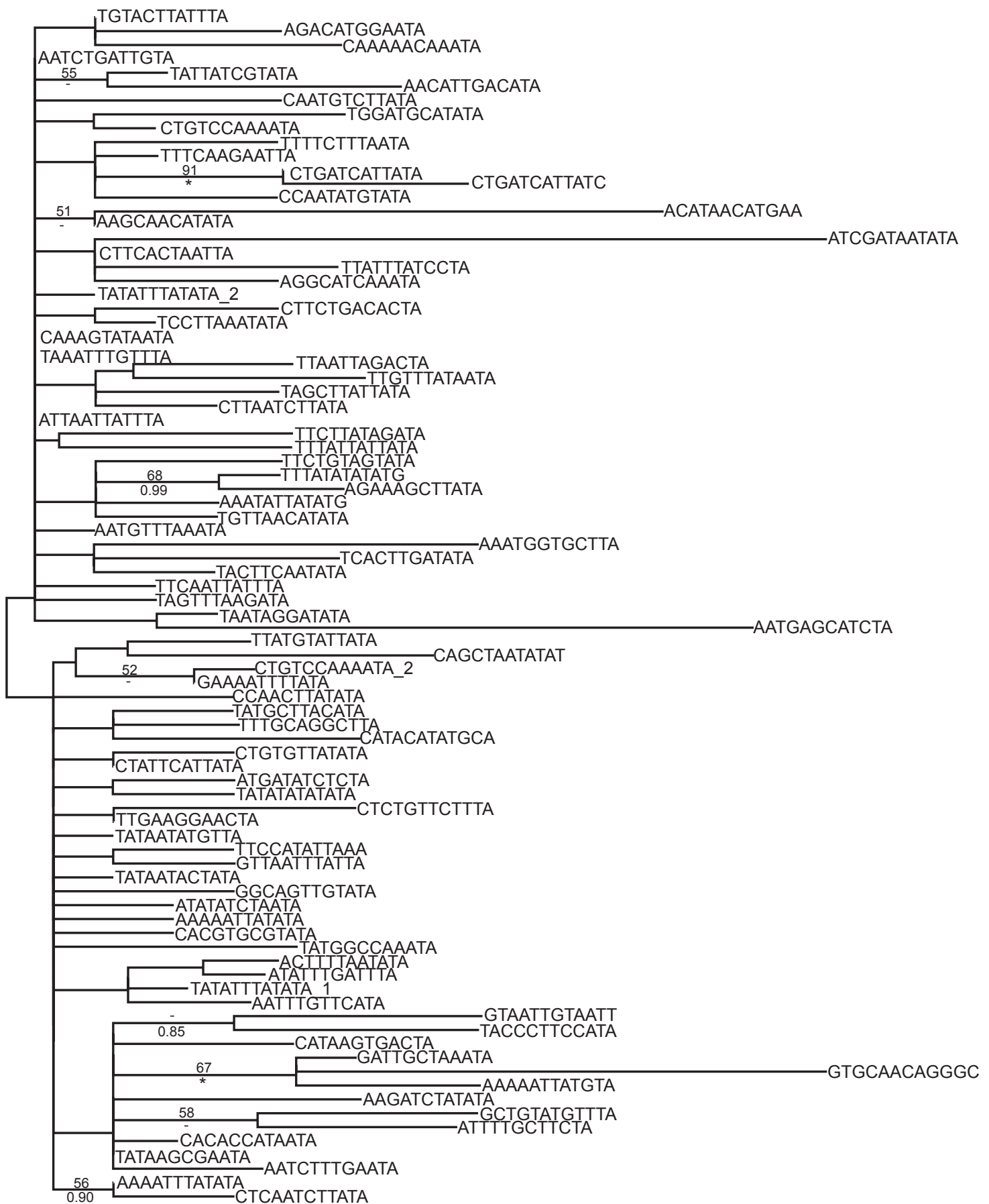

(A) *Tdmar2*

0.009

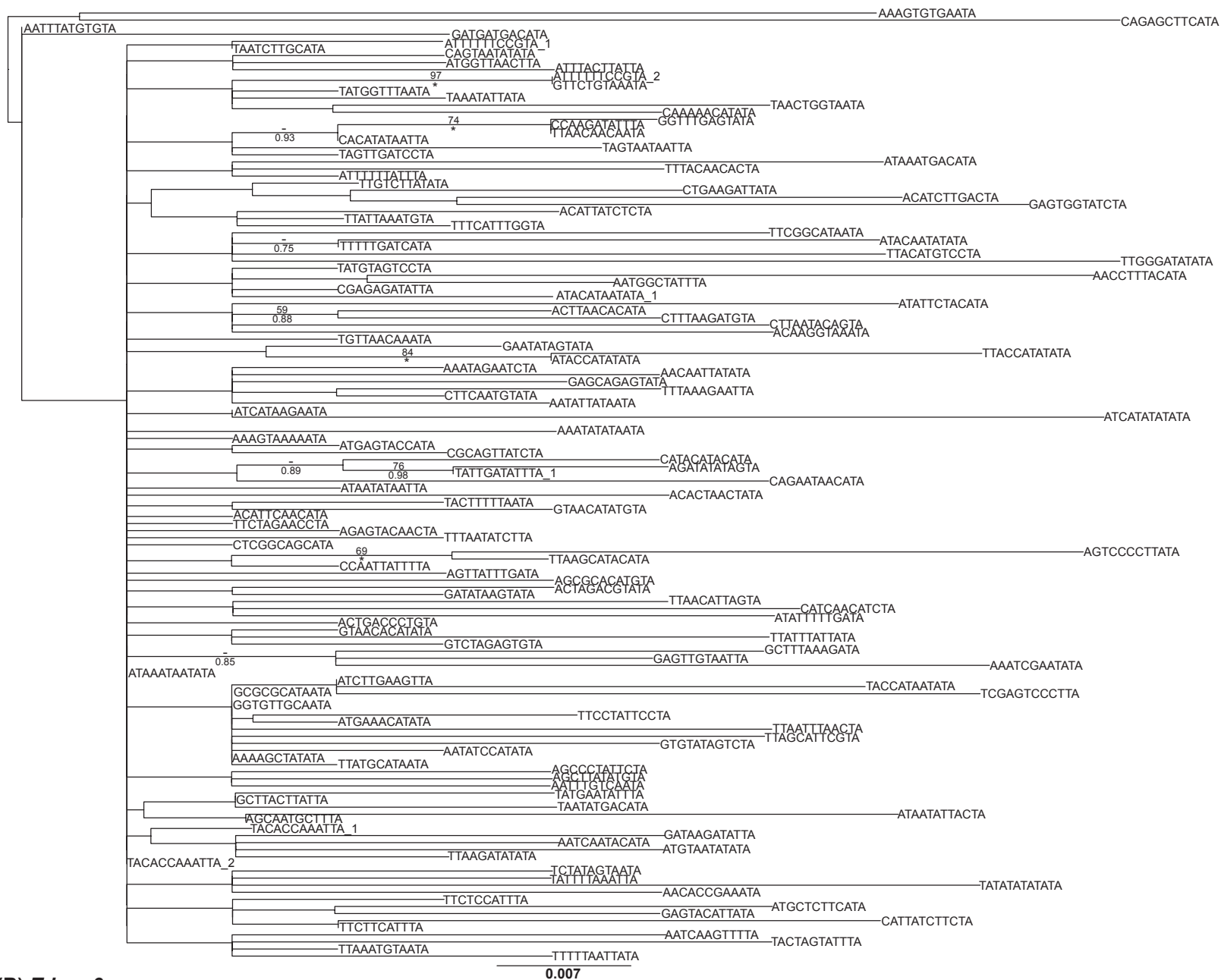

(B) Tdmar3

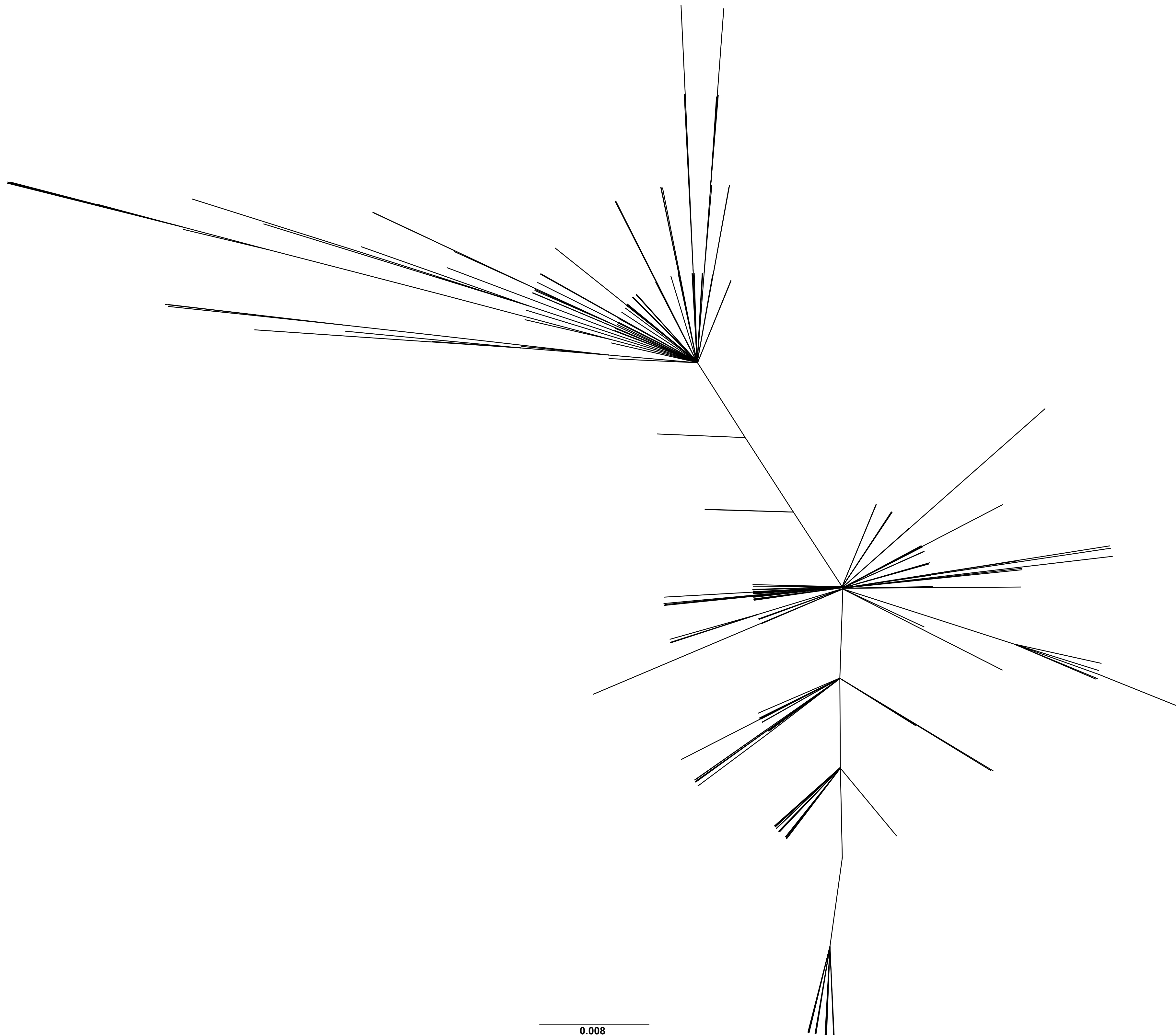

(C) *Tdmar4*

Supplement: S4 Fig — Phylogenies were generated using the GTRCAT model with empirical base frequencies. A) Tdmar2 created from 187 aligned nucleotide positions, B) Tdmar3 created from 204 aligned nucleotide positions, C) Tdmar4 created from 197 aligned nucleotide positions. OTU labels are the 5’ flanking DNA of the ITR for A and B. C is presented as a radial tree and both the support values and OTU labels are omitted due to the large number of sequences. The phylogeny layouts are otherwise the same as in S1 Fig. (PDF) [file pone.0235984.s006.pdf]

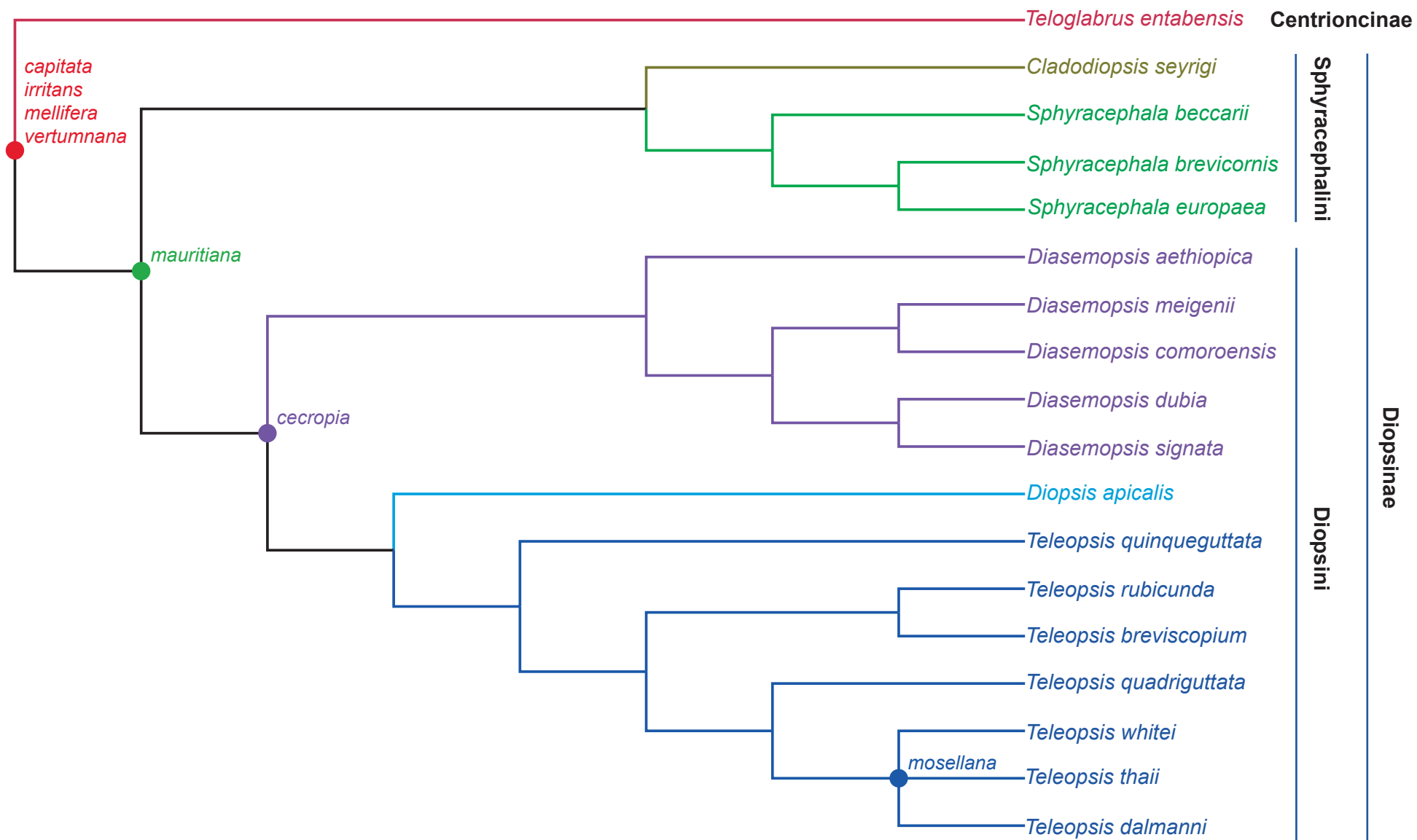

Supplement: S5 Fig — Circles represent the putative origin points of the subfamilies. The tree layout is the same as Fig 1. (PDF) [file pone.0235984.s007.pdf]
