# Supplementary material for: The evolutionary history of mariner elements in stalk-eyed flies reveals the horizontal transfer of transposons from insects into the genome of the cnidarian Hydra vulgaris
Source: PLoS One. 2020 Jul 13;15(7):e0235984. doi: 10.1371/journal.pone.0235984 (PMC7357744; doi:10.1371/journal.pone.0235984)
Supplement: S1 Table — (DOCX) [file pone.0235984.s008.docx]

**S1 Table**. *mariner* sequences generated in this study through PCR amplification.

| **Species** | ***capitata*** | ***irritans*** | ***mauritiana*** | ***mellifera*** | ***vertumnana*** |
| --- | --- | --- | --- | --- | --- |
| *Te. entabensis* | *Temar4* (NA)  MN719940 | *Temar2.1* (NA)  MN719934  *Temar2.2* (Au)  MN719935  *Temar2.3* (NA)  MN719936  *Temar2.4* (Au)  MN719937 |  | *Temar3.1* (Au)  MN719938  *Temar3.2* (NA)  MN719939 | *Temar1.1* (Au)  MN719928  *Temar1.2* (NA)  MN719929  *Temar1.3* (Au)  MN719930  *Temar1.4* (NA)  MN719931  *Temar1.5* (NA)  MN719932  *Temar1.6* (Au)  MN719933 |
| *D. aethiopica* |  | *Damar3.1* (NA)  MN719923  *Damar3.2* (Au)  MN719924 | *Damar1.1* (NA)  MN719915  *Damar1.2* (NA)  MN719916  *Damar1.3* (NA)  MN719917  *Damar1.4* (NA)  MN719918  *Damar1.5* (NA)  MN719919  *Damar1.6* (NA)  MN719920  *Damar1.7* (NA)  MN719921 | *Damar2* (NA)  MN719922 |  |
| *Di. apicalis* |  |  |  |  | *Dioamar1.1* (Au)  MN719925  *Dioamar1.2* (NA)  MN719926  *Dioamar1.3* (Au)  MN719927 |

**Note**: NA – non-autonomous element. Au – putatively autonomous element
