# Supplementary material for: The evolutionary history of mariner elements in stalk-eyed flies reveals the horizontal transfer of transposons from insects into the genome of the cnidarian Hydra vulgaris
Source: PLoS One. 2020 Jul 13;15(7):e0235984. doi: 10.1371/journal.pone.0235984 (PMC7357744; doi:10.1371/journal.pone.0235984)
Supplement: S2 Table — (DOCX) [file pone.0235984.s009.docx]

**S2 Table**. Number of RNA-Seq reads mapped to *mariner* *tnpase* sequences

| ***mariner* Subfamily** | **Length of CDS** | **No. of Reads** | | **Transcripts Per Million** |
| --- | --- | --- | --- | --- |
|  |  | **Absolute Number** | **Percentage of Total Reads** |  |
| *Tdmar1.4*: Male Head | 482 | 140 | 0.0002% | 0.3802 |
| *Tdmar2*: Male Head | 1038 | 3,271 | 0.0051% | 4.1244 |
| *Tdmar3*: Male Head | 1050 | 2,507 | 0.0039% | 3.1250 |
| *Tdmar4*: Male Head | 1077 | 21,423 | 0.0334% | 26.0342 |
| *Tdmar1.4*: Female Head | 482 | 233 | 0.0002% | 0.2729 |
| *Tdmar2*: Female Head | 1038 | 2,641 | 0.0004% | 3.2183 |
| *Tdmar3*: Female Head | 1050 | 2,055 | 0.0031% | 2.4756 |
| *Tdmar4*: Female Head | 1077 | 19,493 | 0.0294% | 22.8938 |
| *Tdmar1.4*: Testes | 482 | 171 | 0.0005% | 1.0185 |
| *Tdmar2*: Testes | 1038 | 461 | 0.0013% | 1.2750 |
| *Tdmar3*: Testes | 1050 | 836 | 0.0024% | 2.2857 |
| *Tdmar4*: Testes | 1077 | 4,294 | 0.0123% | 11.4460 |
| *Tdmar1.4*: Ovaries | 482 | 140 | 0.0004% | 0.7941 |
| *Tdmar2*: Ovaries | 1038 | 2,449 | 0.0067% | 6.4507 |
| *Tdmar3*: Ovaries | 1050 | 1,325 | 0.0036% | 3.4502 |
| *Tdmar4*: Ovaries | 1077 | 3,729 | 0.0102% | 9.4666 |
| *Tdmar1.4*: Larvae | 482 | 233 | 0.0005% | 1.0777 |
| *Tdmar2*: Larvae | 1038 | 7,134 | 0.0159% | 15.3229 |
| *Tdmar3*: Larvae | 1050 | 4,691 | 0.0105% | 9.9605 |
| *Tdmar4*: Larvae | 1077 | 17,358 | 0.0387% | 35.9327 |
| *Twmar1.2*: Testes | 507 | 84 | 0.0001% | 0.2479 |
| *Twmar2*: Testes | 1038 | 1,939 | 0.0029% | 2.7948 |
| *Twmar3*: Testes | 1050 | 1,922 | 0.0028% | 2.7386 |
| *Twmar4*: Testes | 1077 | 15,061 | 0.0223% | 20.9222 |
| *Tqmar1.2*: Testes | 586 | 305 | 0.0002% | 0.2433 |
| *Tqmar2.2*: Testes | 453 | 1 | <0.0001% | 0.0010 |
| *Tqmar3*: Testes | 474 | 30 | <0.0001% | 0.0296 |
| *Tqmar4*: Testes | 441 | 8 | <0.0001% | 0.0085 |
| *Hvmar1*: Head | 1065 | 961 | 0.0018% | 1.6639 |
| *Hvmar2*: Head | 1038 | 203 | 0.0004% | 0.3606 |
| *Hvmar1*: Tentacle Head | 1065 | 1,172 | 0.0017% | 1.6464 |
| *Hvmar2*: Tentacle Head | 1038 | 134 | 0.0002% | 0.1931 |
| *Hvmar1*: Whole Polyp | 1065 | 1,174 | 0.0026% | 2.4512 |
| *Hvmar2*: Whole Polyp | 1038 | 185 | 0.0004% | 0.3963 |
| *Hvmar1*: Body Column | 1065 | 2,433 | 0.0043% | 4.0553 |
| *Hvmar2*: Body Column | 1038 | 314 | 0.0006% | 0.5370 |
| *Hvmar1*: Foot | 1065 | 318 | 0.0006% | 0.5912 |
| *Hvmar2*: Foot | 1038 | 65 | 0.0001% | 0.1240 |
