# Supplementary material for: The evolutionary history of mariner elements in stalk-eyed flies reveals the horizontal transfer of transposons from insects into the genome of the cnidarian Hydra vulgaris
Source: PLoS One. 2020 Jul 13;15(7):e0235984. doi: 10.1371/journal.pone.0235984 (PMC7357744; doi:10.1371/journal.pone.0235984)
Supplement: S3 Table — (DOCX) [file pone.0235984.s010.docx]

**S3 Table.** SRA RNA-Seq files used in gene expression analyses.

| **SRA File** | **Tissue** | **No. of Reads** |
| --- | --- | --- |
| **Diopsidae** |  |  |
| ***T. dalmanni*** |  |  |
| SRX481937 | Male Head | 64,180,074 |
| SRX481966 | Female Head | 66,408,686 |
| SRX481974 | Testes | 34,833,194 |
| SRX481976 | Ovaries | 36,574,910 |
| SRX481981 | Larvae | 44,853,242 |
| ***T. quinqueguttata*** |  |  |
| SRX1490590 | Testes | 84,728,454 |
| SRX1490591 | Testes | 84,251,682 |
| ***T. whitei*** |  |  |
| SRX485305 | Testes | 67,507,652 |
| **Hydridae** |  |  |
| ***H. vulgaris*** |  |  |
| SRX4916622 | Head | 54,231,932 |
| SRX4916623 | Tentacle | 67,507,652 |
| SRX4916624 | Whole Polyp | 44,970,962 |
| SRX4916625 | Body Tissue | 56,333,708 |
| SRX4916626 | Foot | 50,506,116 |
